# A Circuit for Integration of Head- and Visual-Motion Signals in Layer 6 of Mouse Primary Visual Cortex

**DOI:** 10.1016/j.neuron.2018.02.023

**Published:** 2018-04-04

**Authors:** Mateo Vélez-Fort, Edward F. Bracey, Sepiedeh Keshavarzi, Charly V. Rousseau, Lee Cossell, Stephen C. Lenzi, Molly Strom, Troy W. Margrie

**Affiliations:** 1 The Sainsbury Wellcome Centre for Neural Circuits and Behaviour, University College London, 25 Howland Street, London W1T 4JG, UK

**Keywords:** mouse primary visual cortex, head-velocity signals, layer 6 neurons, egocentric framework, Bayesian approach, Neuropixels dense silicon probe, whole-cell patch clamp, 2P imaging, go/no go task

## Abstract

To interpret visual-motion events, the underlying computation must involve internal reference to the motion status of the observer’s head. We show here that layer 6 (L6) principal neurons in mouse primary visual cortex (V1) receive a diffuse, vestibular-mediated synaptic input that signals the angular velocity of horizontal rotation. Behavioral and theoretical experiments indicate that these inputs, distributed over a network of 100 L6 neurons, provide both a reliable estimate and, therefore, physiological separation of head-velocity signals. During head rotation in the presence of visual stimuli, L6 neurons exhibit postsynaptic responses that approximate the arithmetic sum of the vestibular and visual-motion response. Functional input mapping reveals that these internal motion signals arrive into L6 via a direct projection from the retrosplenial cortex. We therefore propose that visual-motion processing in V1 L6 is multisensory and contextually dependent on the motion status of the animal’s head.

## Highlights

• Inputs onto V1 L6 neurons convey head-motion information
• These L6 signals are widespread and provide a reliable estimate of angular velocity
• In L6 neurons, vestibular and visual inputs sum during sensory processing
• At least in part, V1 L6 head-motion signals are conveyed via an RSP-V1 pathway

## Teaser

V1 layer 6 neurons receive a widespread head-motion signal that is integrated with visual input during visual-motion processing. These V1 head-motion inputs project from the retrosplenial cortex, a multisensory area involved in spatial navigation and contextual processing.

## Introduction

Throughout the visual system, the activity of individual neurons signals specific features of object motion (Barlow and Levick, 1965, Dräger, 1975, Hubel, 1960, Hubel and Wiesel, 1959, Hubel and Wiesel, 1968, Niell, 2015, Van Essen and Gallant, 1994). However, to detect the motion trajectory and speed of an object in the external world, visual stimuli must be placed within a context that includes information regarding the motion status of the observer. This integrative process is fundamental, since it underlies judgments of spatiotemporal coincidence (Hoy et al., 2016), including the distance (Barlow et al., 1967), direction (Hubel and Wiesel, 1959, Hubel and Wiesel, 1968), and speed (Roth et al., 2012, Van Essen and Gallant, 1994) of potential animate activity (Grossman and Blake, 2002) that may indicate the presence of a threat, food source, or conspecific.

Growing evidence suggests that attention (Niell and Stryker, 2010, Roelfsema et al., 1998, Zhang et al., 2014), learning (Makino and Komiyama, 2015, Poort et al., 2015, Yan et al., 2014), and context (Fiser et al., 2016, Roth et al., 2016) modulate activity in the primary visual cortex (V1). For example, in head-fixed animals, running evokes modulation of V1 activity (Erisken et al., 2014, Fu et al., 2014, Niell and Stryker, 2010, Polack et al., 2013, Saleem et al., 2013), and in virtual-reality environments where visual flow can be uncoupled from running speed, V1 activity can also report sensory-motor mismatch when the actual and expected visual flow are not coherent (Keller et al., 2012).

More broadly, internally generated signals are believed to play a fundamental role in spatial sensory processing and navigation (Morris et al., 1982, Tsoar et al., 2011). The neuronal underpinnings of these representations are widely distributed (Moser et al., 2008) and, at least in part, utilize internal vestibular signals (Cho and Sharp, 2001, Smith et al., 2005, Stackman et al., 2002, Taube et al., 1990, Valerio and Taube, 2016). While the involvement of vestibular signaling in limbic regions known to contribute to spatial processing is well described, the extent to which this internal signal is involved in primary sensory cortical representation is not known.

Stimulation of the vestibular organ or nerve does, however, evoke responses in V1 (Grüsser and Grüsser-Cornehls, 1972, Rancz et al., 2015, Vanni-Mercier and Magnin, 1982) and in the retrosplenial cortex (RSP) (Rancz et al., 2015), a multisensory area known to project directly to V1 (Leinweber et al., 2017, Makino and Komiyama, 2015, van Groen and Wyss, 1992, Vélez-Fort et al., 2014, Vogt and Miller, 1983). From a functional perspective, the RSP appears well positioned to convey head-motion information to V1, since it receives projections from subcortical vestibular-related nuclei (Amin et al., 2010, Oh et al., 2014, Shibata, 1993, Van Groen and Wyss, 1995, Van Groen and Wyss, 2003, Vogt et al., 1981) and signals running speed, head direction, and angular velocity (Cho and Sharp, 2001). Although the functional relevance of the RSP projection into V1 remains unknown, its dominance in the presynaptic network (Leinweber et al., 2017, Vélez-Fort et al., 2014) and role in visual-based spatial navigation (Alexander and Nitz, 2015, Chen et al., 1994, Jacob et al., 2017, Mao et al., 2017, Vann et al., 2009) make it an attractive candidate for providing internal motion signals.

## Results

### Head-Motion Signals in Deep Layers of V1

**Figure 1:**
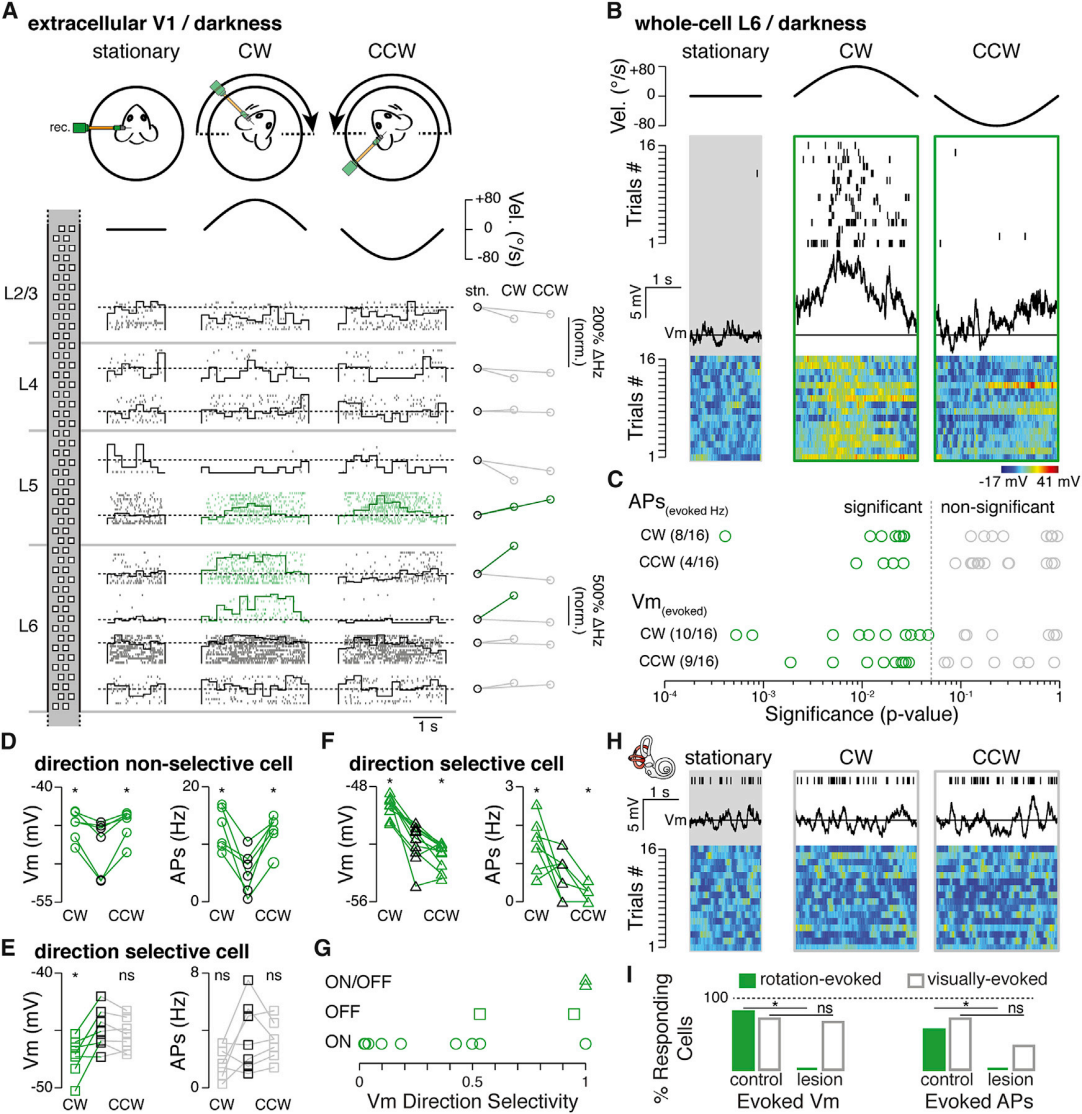
Head-Rotation-Evoked Responses in V1 L6 Neurons in Awake Mice (A) Top: schematic of the recording setup and velocity profile of the rotation stimulus (black trace). Left: schematic of the dense extracellular recording probe, highlighting the location of individual recording channels located in specific cortical layers. Middle: example raster plots obtained from units of V1 neurons recorded in the absence (stationary) and presence of passive rotation (CW and CCW). Overlaid: peri-stimulus time histograms corresponding to each raster (bin size = 250 ms). Dashed line indicates the average spike count per bin during baseline. Right: normalized percentage change in firing rate compared to spontaneous firing when the platform is stationary (stn). Highlighted in green are isolated units that show a significant change in rotation-evoked responses in either or both directions (significance threshold p = 0.05, Wilcoxon signed-rank test). (B) Top: velocity profile of the rotation stimulus (black trace). Middle: raster plot of spiking obtained from a whole-cell recording of one L6 neuron in the absence and presence of head rotation. Below: trace showing the average membrane potential (V_m_) recorded in the same cell. Horizontal line indicates the mean membrane potential recorded in the absence of rotation. Bottom: false color raster plot indicating the change in membrane potential for 16 consecutive trials. (C) Logarithmic plot of p values for spike rate and membrane potential (V_m_) responses recorded in the absence versus the presence of rotation (n = 16 cells, 32 directions). Dashed line is the statistical significance cut-off (p = 0.05, Wilcoxon signed-rank test). (D–F) Left: plot of the average membrane potential recorded during baseline (black symbols) and rotation (CW and CCW) for ON (D; n = 6 trials), OFF (E; n = 7 trials), and ON/OFF (F; n = 10 trials) responding neurons. Right: plot of the spike rate per trial for the same three cells. Star indicates statistical significance (significance threshold p = 0.05, Wilcoxon signed-rank test). (G) Plot of the rotation direction selectivity scores for ON (circle), OFF (squares), and ON/OFF (triangles) neurons (n = 13 cells). (H) Top: raster plot obtained from one L6 neuron recorded in a bilaterally vestibular-lesioned mouse during the absence and presence of rotation (action potentials recorded for all 16 trials are displayed as a single raster). Middle: a trace of the average membrane potential (V_m_) recorded in the same cell. Horizontal line indicates the mean membrane potential recorded in the absence of rotation. Below: false color raster plot indicating the change in membrane potential recorded for 16 consecutive trials. Color scale is same as shown in (B). (I) Proportion of L6 cells responding to rotation (control n = 16, lesioned n = 7 cells) and visual stimulation (control n = 7, lesioned n = 6 cells). Star indicates statistical significance (significance threshold p = 0.05, Fisher exact test). See also Figures S1–S3.

To investigate whether V1 has access to head-motion signals, we performed extracellular and whole-cell recordings in the left brain hemisphere of awake, head-fixed mice during passive rotation in complete darkness (Arenz et al., 2008) (Figure 1A). First, using a dense silicon probe that enables us to record simultaneously across all cortical layers (Jun et al., 2017), we found that well-isolated units (n = 28 units, n = 3 mice) located in layers 5 (L5) and 6 were most likely to show rotation-evoked activity (L5, 2/7; L6, 9/14; compared to L4, n = 1/7, significance threshold p = 0.05, Wilcoxon signed-rank test; Figure 1A). The inability to isolate units in L2/3 (Saleem et al., 2013) motivated us to confirm these initial data using whole-cell recordings. In line with the extracellular data, while rotation-evoked action potential activity was not observed in L2/3 regular spiking neurons (L2/3, 0/8, significance threshold p = 0.05, Wilcoxon signed-rank test), responses were observed in deep-layer principal cells (L5, 2/5 cells; L6, 9/16 cells; significance threshold p = 0.05, Wilcoxon signed-rank test). Taken together, both extra- and intra-cellular recording approaches showed that deep-layer V1 neurons receive and broadcast a head-motion signal.

Given the known RSP projection onto V1 L6 (Vélez-Fort et al., 2014) and the large fraction of cells that responded to rotation, we focused on L6 neurons to investigate the underlying synaptic properties of these responses. First, the membrane potential of nearly all L6 cells (n = 13/16 cells, significance threshold p = 0.05, Wilcoxon signed-rank test; Figures 1B and 1C) was found either to significantly depolarize or to hyperpolarize when the animal was rotated in either the clockwise (CW, n = 10/16 cells) or counter-clockwise (CCW; n = 9/16 cells) direction. The overall average of the peak amplitude of the response was 11.28 ± 1.12 mV (n = 13 cells, n = 19 directions). The overall mean membrane potential response calculated across the entire rotation trajectory (−90° to +90° or vice versa) was 1.80 ± 0.35 mV. Since the probability of observing responses in cortico-thalamic (CT) and cortico-cortical (CC) neurons was similar (CT, 10/13 cells versus CC, 3/3 cells; p = 1, Fisher exact test; STAR Methods), these two cell types were pooled for the remainder of the analysis.

Qualitatively, a small fraction of cells showed tonic membrane potential depolarizations during rotation. In the majority of cases, however, the membrane potential of cells appeared to respond according to the speed of rotation. Overall, these responses fell into three general types: first, “ON responses” were characterized by a synaptic depolarization and often firing of action potentials during rotation in either (selective) or both (non-selective) directions (n = 9/13 cells; Figure 1D). Second, “OFF responses” showed a hyperpolarization of the membrane potential in either or both directions (n = 2/13 cells; Figure 1E), while the third category of responses was characterized by a significant membrane potential depolarization in one direction and a hyperpolarization in the other, and was thus classified as direction-specific “ON/OFF responses” (n = 2/13 cells; Figure 1F). At the population level, rotation-evoked synaptic input onto L6 cells appeared widespread and showed a broad range of selectivity to the direction of rotation (Figure 1G).

To determine the sensory origin of these L6 inputs, we bilaterally perturbed the posterior and horizontal semi-circular canals and injected the ototoxic antibacterial agent kanamycin. Lesioning the canals produced a circling behavioral phenotype consistent with vestibular dysfunction (Valerio and Taube, 2016, Vidal et al., 2004) (Figures S1A and S1B) and the complete loss of rotation-evoked subthreshold responses (control, n = 19/32 directions versus lesioned, n = 0/14 directions; n = 7 cells [6 CT and 1 CC], p = 5 × 10^−4^, Fisher exact test; Figure 1H), while responses to visual stimuli remained comparable to non-lesioned mice (mean membrane potential response calculated across the entire rotation trajectory from baseline, control, 2.33 ± 0.26 mV versus lesioned, 3.18 ± 0.67 mV; p = 0.46, Wilcoxon rank-sum test; Figures 1H and 1I).

To explore the possibility that changes in eye position could account for these observed rotation-evoked responses (Toyama et al., 1984), we also performed event-triggered averaging of the membrane potential while monitoring the position of both eyes under infrared illumination. We found no correlative evidence for eye position-related L6 membrane potential responses (n = 349 temporal-medial events, n = 192 medial-temporal events, n = 3 cells, significance threshold p = 0.05, Wilcoxon signed-rank test; Figures S2A and S2B). Furthermore, we found no correlative evidence for spontaneous eye position-related membrane potential responses (n = 6 temporal to medial events, n = 3 cells, significance threshold p = 0.05, Wilcoxon signed-rank test). In addition, peri-ocular injections of Lidocaine that reduced eye mobility did not impact either the likelihood of observing evoked responses (n = 3/3 cells, p = 1, Fisher exact test; Figures S3A–S3C) or the mean membrane potential response calculated across the entire rotation trajectory (control, 1.80 ± 0.35 mV, n = 19 directions versus Lidocaine, 1.65 ± 0.49 mV, n = 4 directions; p = 0.78, Wilcoxon rank-sum test; Figure S3C). We therefore included these cells for all subsequent analysis. Taken together, these data show that these membrane potential responses in V1 L6 neurons are not due to changes in eye position but instead arise from sensory transduction in the vestibular apparatus, and can be attributed to the motion of the head during passive rotation.

### V1 L6 Neurons Receive Angular Velocity Signals

**Figure 2:**
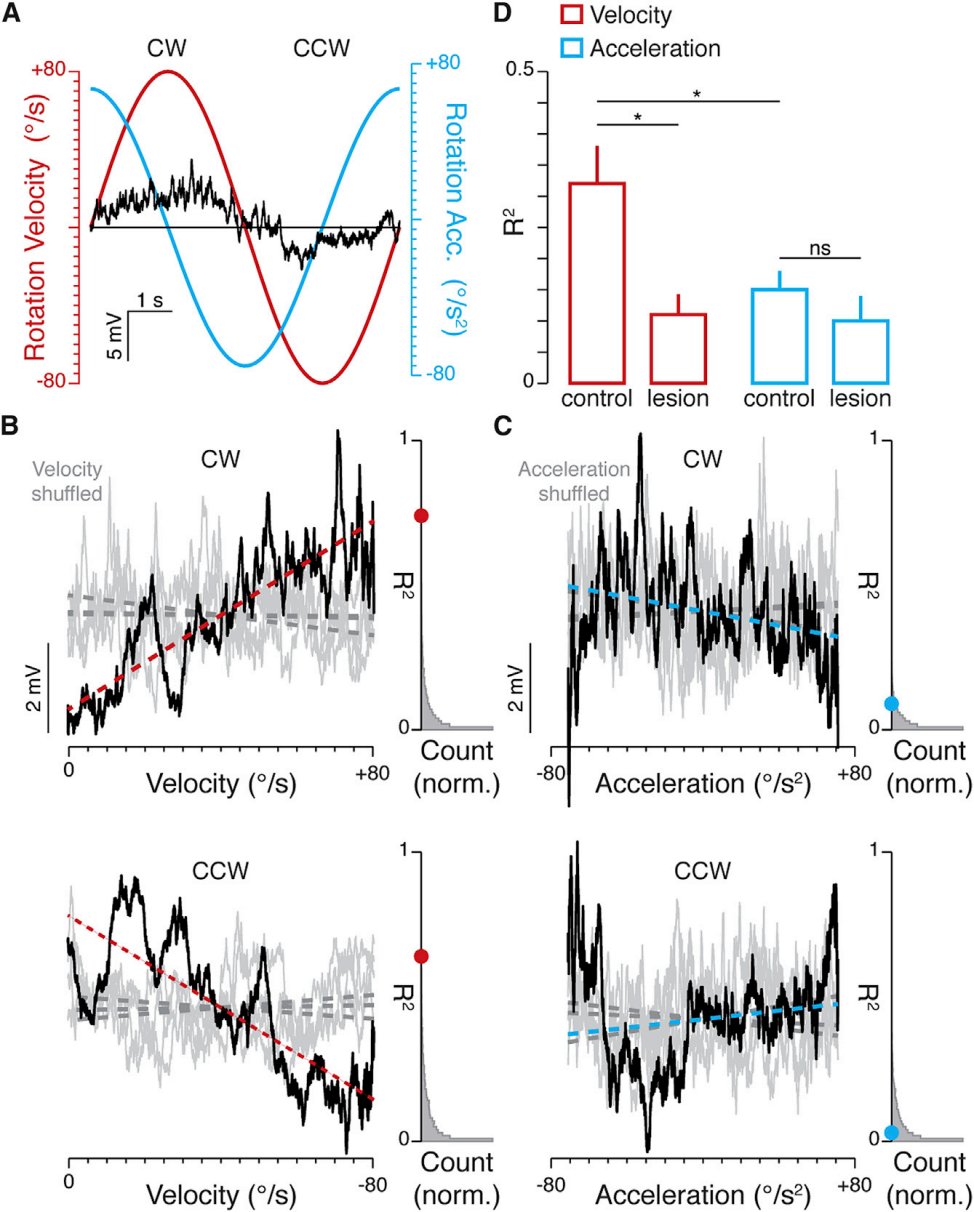
Head-Velocity Signals in V1 L6 Neurons (A) Velocity (red line) and acceleration (blue line) profile of the rotation stimulus and the evoked membrane potential (average of 10 trials, black), recorded from a direction-selective cell. Horizontal line indicates the mean membrane potential recorded in the absence of rotation. (B and C) The average membrane potential (black trace) of the recorded cell shown in (A), plotted against rotation velocity (B) and acceleration (C) for CW (top) and CCW (bottom) directions (resampled velocity = 0.008°/s; acceleration = 0.007°/s^2^). Gray traces are examples of the membrane potential when shuffled in the velocity (B) and acceleration (C) domains (4 out of 10,000 iterations shown). Overlaid are the linear regression fits of the raw (colored dashed line) and the shuffled (gray dashed lines) membrane potential. Gray histograms represent the distributions of the R^2^ determined for all iterations of the shuffled data (10,000 R^2^ values in each direction) compared to the R^2^ of the raw data (red and blue circles) for each direction. (D) Bar graphs of R^2^ values for velocity and acceleration, in control (n = 23 directions) and lesioned (n = 14 directions) animals. Bars and solid lines = mean ± SEM. Star indicates significant differences when comparing experimental conditions (significance threshold p = 0.05, Wilcoxon rank-sum and Wilcoxon sign-rank tests).

To explore the motion-encoding properties of these inputs, we identified all cells where the average amplitude of the postsynaptic membrane potential response changed significantly during rotation in either direction (n = 19 cells, n = 23 directions). For each direction, we then re-sampled the membrane potential data so that each velocity bin (0.008°/s) contained the same number of data points (1 data point per bin; STAR Methods; Figures 2A and 2B). We then performed circular permutations of the angular velocity of each trial (bin size = 0.008°/s, 10,000 iterations per direction) while retaining the temporal structure of the membrane voltage response (Figure 2B). Linear regression analysis was then performed on the “shuffled” and “raw” (non-shuffled) membrane potential data (STAR Methods). In contrast to the shuffled data, we found that the angular velocity was often linearly correlated to the recorded membrane potential. Indeed, in 14/23 cases the calculated R^2^ value for the evoked membrane potential was significantly larger when compared to the distribution of R^2^ values obtained for the shuffled data (significance threshold p = 0.05; Figure 2B) and responses recorded in lesioned animals (control, R^2^ = 0.32 ± 0.06, n = 23 directions versus lesioned, R^2^ = 0.11 ± 0.03, n = 14 directions; p = 0.02, Wilcoxon rank-sum test).

By way of comparison, the R^2^ values determined for angular velocity significantly exceeded those determined for acceleration (velocity, R^2^ = 0.32 ± 0.06, n = 23 directions versus acceleration, R^2^ = 0.15 ± 0.03, n = 23 directions; p = 0.04, Wilcoxon signed-rank test), which were not significantly different between intact and lesioned mice (control, R^2^ = 0.15 ± 0.03, n = 23 directions versus lesioned, R^2^ = 0.10 ± 0.04, n = 14 directions; p = 0.08, Wilcoxon rank-sum test; Figures 2C and 2D). Taken together, these data show that for the majority of V1 L6 neurons showing evoked responses, the membrane potential response indicates the angular velocity of rotation.

### Robust Velocity Coding within L6 Networks

Presumably, the quality and potential physiological significance of these V1 responses would be reflected by their signal-to-noise ratio, which can be quantified using a variety of theoretical approaches, such as mutual information and Bayes’ theorem (Arenz et al., 2008, Thiel et al., 2007). However, in the absence of directly relevant behavioral data, theoretical estimates are difficult to interpret. If V1 networks use these inputs to generate a comprehensive and integrative map of internal and external motion signals, we argued (1) that the theoretical resolution of these physiological responses should approach the psychophysical limits of the animal’s ability to distinguish “self-motion” and (2) that realistic-sized networks of L6 neurons should be able to extract angular velocity information on a moment-to-moment basis.

**Figure 3:**
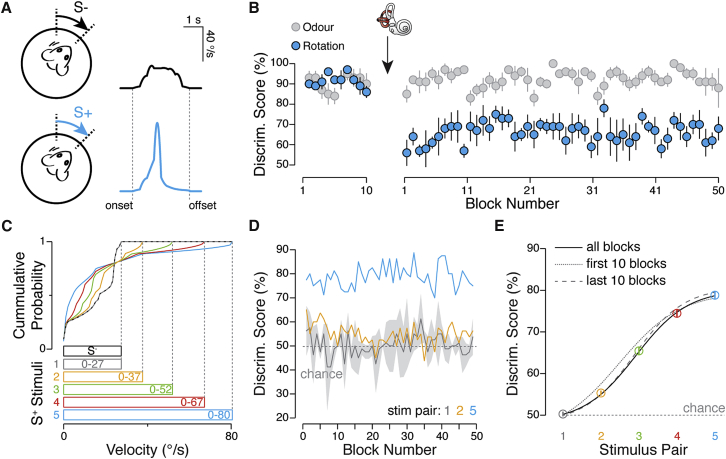
Rotation Discrimination in Mice (A) Stimulus used for training mice to learn to discriminate rotation. Rotation stimuli are presented with the same onset and offset times, but different velocity profiles (S^−^ stimulus range, 0–27°/s, black; S^+^ stimulus range, 0–80°/s, blue). (B) Discrimination accuracy scores for rotation stimuli (blue circles) interleaved with odor stimuli (light gray circles) for five mice, before and after vestibular lesion (black arrow). Circles and solid lines = mean ± SEM. (C) Cumulative probability plot of the velocity used for all stimuli in the behavioral discrimination task. Below: bar graph shows the velocity range for the S^−^ and all the S^+^ stimuli. (D) Average discrimination performance (per block) of four mice plotted for S^−^/S^+^1 (gray trace = mean ± SD), S^−^/S^+^2 (orange), and S^−^/S^+^5 (blue) stimulus pairs. (E) Average discrimination scores for all stimulus pairs and all blocks (color circles = mean ± SEM). Overlaid are the sigmoid fits for the psychometric curve for all stimulus pairs for the first ten (thin dashed line), the last ten (thick dashed line), and all fifty blocks (black line). See also Figure S4.

To begin to investigate the fidelity of these V1 responses, we trained head-fixed mice in total darkness on a go/no-go task (n = 5 mice) requiring them to discriminate passive rotations whereby a non-rewarded rotation stimulus (S^−^, range = 0–27°/s) was paired with a rewarded stimulus with a larger peak velocity (S^+^, range = 0–80°/s; Figures 3A and S4A). Using this stimulus pair, we found that mice could, above chance, accurately and reliably discern the speed of horizontal rotation (Figure 3B). To assess the contribution of vestibular signaling, we performed bilateral lesions of the posterior and horizontal semi-circular canals and injected the ototoxic antibacterial agent kanamycin. After recovery, animals’ rotation discrimination performance fell to almost chance levels for the same pre-treatment stimulus pair (average discrimination score last 10 blocks pre-treatment, 91.4% ± 0.9% accuracy versus average first 10 blocks post-treatment, 62.3% ± 1.9% accuracy; p < 10^−6^, one-way ANOVA with Bonferroni correction; Figure 3B). In contrast, the same mice continued to reliably discriminate odor stimuli that we interleaved with the rotation stimuli (last 10 blocks pre-treatment, 90.5% ± 1.3% accuracy versus first 10 blocks post-treatment, 93.0% ± 1.1% accuracy; p = 1, one-way ANOVA with Bonferroni correction; Figure 3B), indicating that, while vestibular lesions appeared not to impact general motivation or attention, rotation discrimination performance strongly relied on the vestibular organ.

In another set of experiments (n = 4 mice), once mice reached criterion performance levels for discrimination, we reduced the velocity peak amplitude of the S^+^ without altering the times of motion onset, peak velocity, or offset (Figures 3C, S4B, and S4C). We found that mice could perform above chance when the difference in the velocity ranges of two stimuli exceeded 24°/s (average discrimination scores of last 10 blocks, S^−^/S^+^1 (i.e., identical stimuli), 49.8% ± 0.8% versus S^−^/S^+^3, 65.5% ± 1.9%, p < 10^−6^; S^−^/S^+^4, 76.3% ± 2.1%, p < 10^−6^; S^−^/S^+^5, 79.3% ± 2.2%, p < 10^−6^; one-way ANOVA with Bonferroni correction; Figures 3D and 3E). In contrast, rotation stimuli that differed in their velocity range by only 10°/s (S^−^/S^+^2) could not be reliably distinguished during the equivalent training period (last 10 blocks, S^−^/S^+^1, 49.8% ± 0.8% versus S^−^/S^+^2, 55.4% ± 1.3%; p = 0.24, one-way ANOVA with Bonferroni correction; Figures 3D and 3E). Taken together, these data indicate that internal representations of head motion in mice permit detection and discrimination of head movements, and that the differential threshold for horizontal rotation is approximately 10°/s.

**Figure 4:**
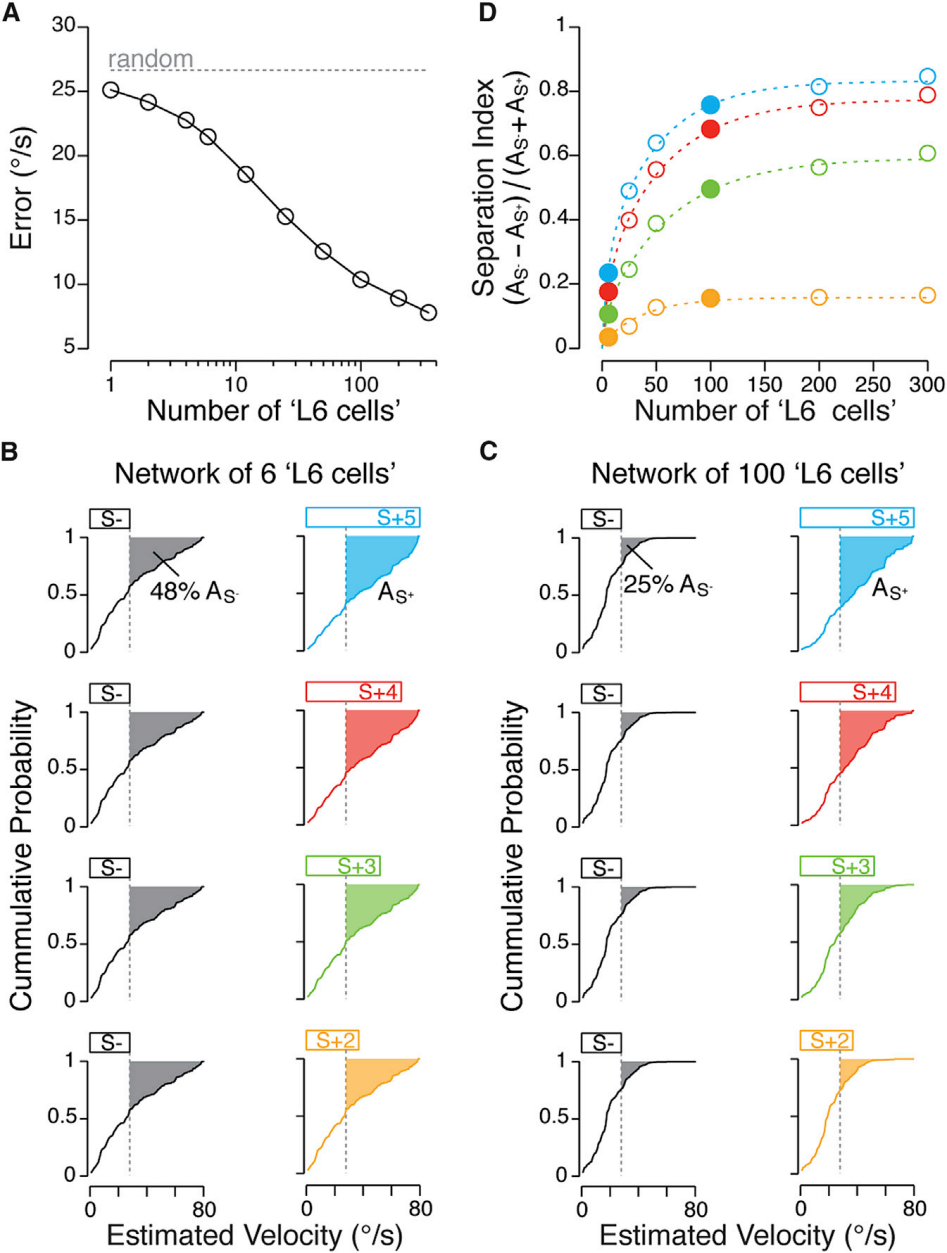
Estimation and Separation of Rotation Velocity by V1 L6 Networks (A) Plot of the difference between the predicted and the actual angular velocity (error) of rotation for inputs distributed between 1 and 348 “cells.” Dashed line indicates performance when making a random prediction at each velocity. (B and C) Left: cumulative distributions of predicted velocities for the S^−^ stimulus for groups of 6 (B) and 100 (C) “cells.” Gray filled area above the curve (A_S_−) indicates the fraction of velocity estimates falling outside the S^−^ velocity range (black horizontal bar). Right: cumulative distributions of predicted velocities for all S^+^. Colored filled area above the curve (A_S_+) indicates the fraction of velocity estimates that fall outside the S^−^ velocity range for each S^+^ (color horizontal bars). (D) Separation index scores for the range of velocities used for different discrimination stimuli (color) for 6–300 L6 cells (circles). Overlaid are the double exponential fits of the separation index scores (dashed lines). The separation index scores computed from (B) and (C) are highlighted (filled circles).

We then argued that, if vestibular-mediated inputs onto V1 L6 cells present a physiologically meaningful “self-motion” cue for integration with visual information, then input patterns distributed across the L6 network would be expected to permit reliable detection and separation of head velocity. First, based on the whole-cell data, we asked how many cells across the L6 network would be required to faithfully signal head velocity to within psychophysical limits. To address this, we treated each rotation-evoked membrane potential trial (n = 348 trials) as reflecting input onto an individual L6 “cell” (Arenz et al., 2008). Then, by pseudo-randomly selecting responses and applying Bayes’ theorem, we generated membrane potential-based velocity estimates for the rotation stimulus for 1–347 cells (range, 0–80°/s at 1°/s resolution; STAR Methods). This indicated that inputs distributed over as few as 100 L6 cells could provide a faithful estimate (10.4°/s) of the actual head velocity (Figure 4A).

Second, by using the membrane potential-based estimates of rotation velocity, we next asked to what extent networks of L6 cells could distinguish two different yet overlapping ranges of velocity. Using the velocity ranges relevant to stimuli presented in the psychophysics experiments, we found that for a network size of only six L6 neurons, almost half of velocity estimates (27 velocity bins times 348 iterations) were erroneous (4,474/9,396 estimated errors [48%] for the actual velocity range S^−^ = 0–27°/s; Figure 4B). Not surprisingly, for a network of 100 L6 cells the fraction of erroneous estimates for the S^−^ range was substantially less (2,383/9,396 estimated errors [25%], p < 10^−6^, chi-square test; Figure 4C).

We next examined the distribution of velocity estimates for all S^+^ ranges and for 6 versus 100 L6 neurons. For 100 cells, the estimates closely matched the probed range of velocities (Figure 4C) and, due to the relatively small fraction of erroneous estimates on the S^−^ range, a network of 100 cells could separate all the discriminable velocity ranges (proportion of cases in which S^−^ was separated from S^+^3 = 27% [95/348], S^+^4 = 81% [281/348], and S^+^5 = 96% [335/348] compared to S^+^2 = 0%; significance threshold p = 0.05, Fisher exact test; Figure 4C; STAR Methods). In contrast, due to the large fraction of errors on the S^−^ range, input onto only six cells could not reliably discern the S^−^ from any of the S^+^ velocity ranges (Figure 4B). Interestingly, this analytical approach also indicated that input onto as many as 300 L6 neurons would not provide reliable separation of the non-discriminable S^+^2 stimulus pair (Figure 4D). These physiological, behavioral, and theoretical data indicate that V1 L6 neurons receive a robust head-velocity signal that may be used as a reliable internal reference of head motion.

### Integration of Visual and Vestibular-Mediated Signals in V1 L6 Neurons

**Figure 5:**
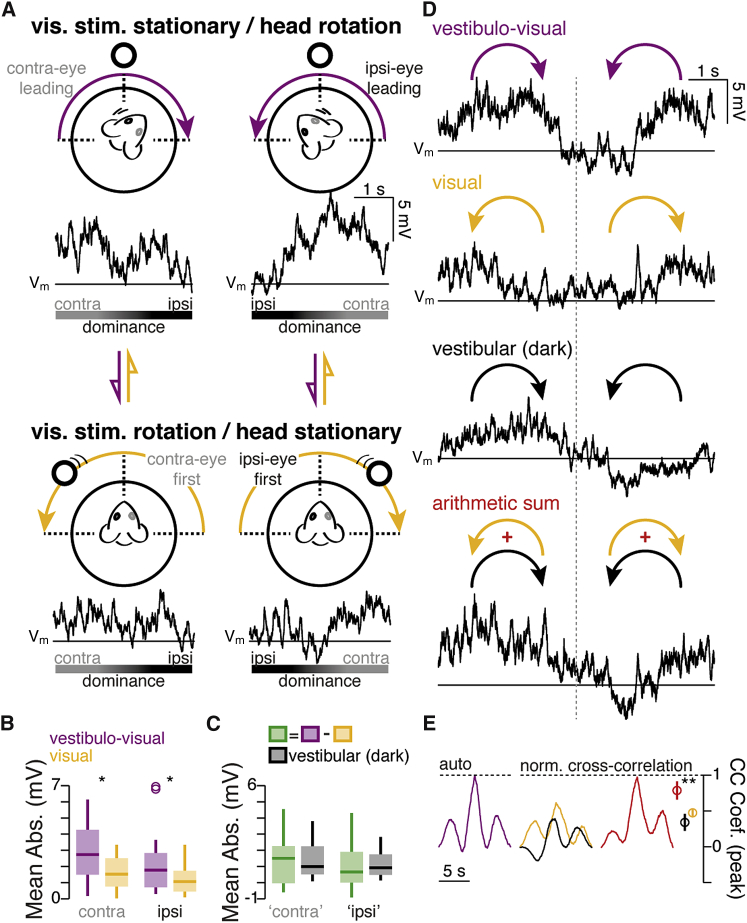
Integration of Head- and Visual-Motion Signals in L6 Neurons (A) Top: schematic showing the experimental design whereby either the mouse is rotated past a stationary visual stimulus (100% contrast white disc, purple arrows) to evoke both vestibular and visual responses, or the visual stimulus is rotated concentrically around the stationary mouse (yellow arrows). For each direction, the first and second halves of the trial were analyzed separately according to the dominance of the visual field (ipsilateral versus contralateral to the recorded left hemisphere). Below schematics: examples of the average membrane potential (V_m_, 10 trials each) recorded from the same cell, as either the animal is rotated past the visual stimulus (top) or the visual stimulus is rotated around the animal (bottom). Horizontal line indicates the mean membrane potential recorded in the absence of rotation. (B) Boxplots of average absolute change in membrane potential (V_m_) recorded for the ipsilateral and contralateral eye fields during rotation of the mouse past the visual stimulus (purple) or rotation of the visual stimulus alone (yellow; n = 14 directions). Star indicates significant differences when comparing the two experimental conditions (significance threshold p = 0.05, Wilcoxon signed-rank test). (C) Boxplots of the difference between the average absolute change in membrane potential (V_m_) evoked by rotation of the mouse past visual stimulus and rotation of the visual stimulus alone (green), versus the average response recorded during rotation in the dark (black). All conditions were recorded within each cell (n = 14 directions). (D) Top: example of the average membrane potential (V_m_) response from another cell recorded during rotation of the mouse past the static visual stimulus for both CW and CCW directions (purple arrows, average from 10 trials each). Middle: average traces showing responses to visual motion when the mouse is static (yellow arrows) and vestibular-evoked responses recorded in darkness (black arrows). Bottom: trace showing the arithmetic sum of the averages of the visual-motion and the dark rotation response. Horizontal lines indicate the mean of the membrane potential recorded in the absence of any rotation. (E) Left: normalized auto-correlogram of the average membrane potential recorded from the cell shown in (D) when rotating the mouse past the visual stimulus (purple). Right: cross-correlograms of the average membrane potential recorded when rotating the mouse past the visual stimulus versus visual stimulus rotation (yellow), rotation in the dark (black), and the arithmetic sum of those responses (red). Circles show the mean normalized (to the auto-correlation peak within cell) peak cross-correlation values (n = 7 cells, mean ± SEM). Star indicates significant differences when comparing the peak values of cross-correlation (significance threshold p = 0.05, Wilcoxon signed-rank test).

To test the idea that head- and visual-motion signals could be integrated in V1 L6, we recorded responses to passive rotation and visual-motion stimulation in the same cell. First, rotation of the mouse past a static visual stimulus (vestibulo-visual stimulus) evoked pronounced membrane potential and spiking responses (n = 7 cells) when compared to recordings during rotation in the dark (mean vestibulo-visual response calculated across the entire rotation trajectory, 2.60 ± 0.49 mV versus vestibular only, 1.21 ± 0.25 mV; n = 14 directions, p = 3.1 × 10^−3^, Wilcoxon signed-rank test). Second, comparison of the vestibulo-visual responses (Figure 5A) to responses recorded when moving the same visual stimulus concentrically in the opposite direction around the static mouse (pure visual-motion stimulus; Figure 5A) produced a reliably different subthreshold response (Figure 5B). Indeed, in both the ipsilateral and contralateral eye fields, the vestibulo-visual responses were significantly more pronounced than pure visual-motion responses (contralateral eye field, mean vestibulo-visual response calculated across the rotation trajectory, 2.89 ± 0.52 mV versus pure visual motion, 1.59 ± 0.28 mV, n = 14 directions, p = 0.01; ipsilateral eye field, vestibulo-visual, 2.35 ± 0.56 mV versus pure visual motion, 1.28 ± 0.27 mV, n = 14 directions, p = 0.01; Wilcoxon signed-rank test; Figure 5B). The difference in the membrane potential amplitude between the vestibulo-visual and the pure visual-motion response was not dissimilar to the amplitude of the response recorded during rotation in darkness (contralateral eye field, difference = 1.30 ± 0.42 mV versus vestibular only = 1.35 ± 0.30 mV, n = 14 directions, p = 0.73; ipsilateral eye field, difference = 1.06 ± 0.39 mV versus vestibular only = 1.09 ± 0.22 mV, n = 14 directions, p = 0.43; Wilcoxon signed-rank test; Figure 5C).

Next, we also examined the temporal properties (i.e., the membrane potential trajectory) of the responses recorded in the same cell under these three experimental conditions (Figure 5D). By taking odd and even trials, we first performed an auto-correlation of the response to the vestibulo-visual stimulus for each cell (Figure 5E). If an evoked response recorded during a different experimental condition was temporally modulated to a similar extent, the two responses would be expected to show a high cross-correlation coefficient. This cross-correlation analysis indicated that vestibulo-visual responses matched most closely to the arithmetic sum of the pure vestibular and the pure visual-motion-evoked responses (normed cross-correlation coefficient for arithmetic sum, 0.79 ± 0.13 versus pure visual motion, 0.47 ± 0.05, p = 0.03; versus vestibular only, 0.34 ± 0.12, p = 0.02; Wilcoxon signed-rank test; Figures 5D and 5E). Taken together, these data show that L6 cells receive head-motion inputs that are functionally distinct from visual inputs. When combined, these signals produce unique patterns of membrane potential activity, which distinguish internal from external motion cues and their combination.

### RSP Is a Source of Internal Motion Signals in V1 L6 Neurons

**Figure 6:**
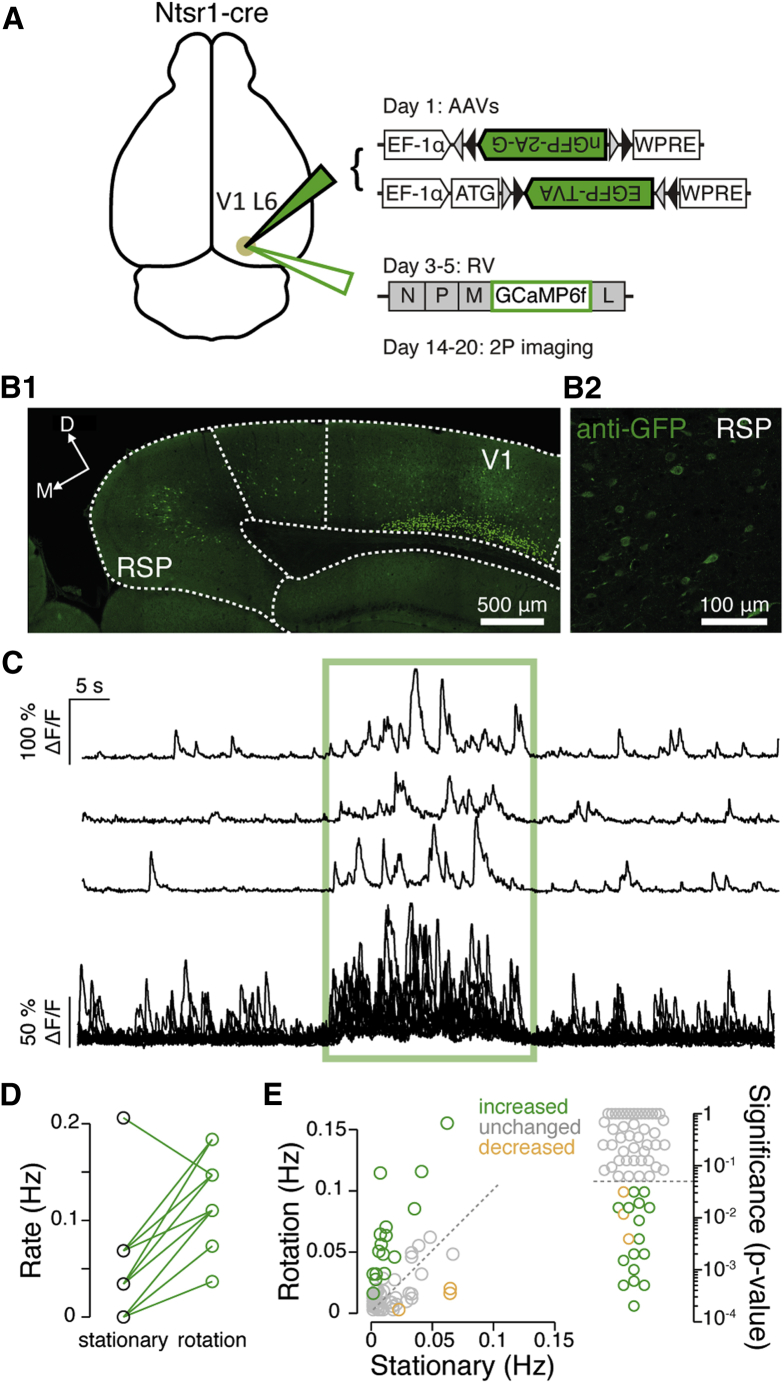
A Head-Motion Signaling Pathway from RSP to V1 L6 (A) Schematic of the helper viruses encoding the rabies G protein and TVA (green), and the modified RV (encoding GCaMP6f) injected into V1 L6 of Ntsr1-cre mice for subsequent imaging in RSP. (B1) Confocal image of a coronal brain slice containing both V1 and the RSP showing the nuclear expression of nGFP in host cells (L6 of V1) and cells expressing GCaMP6f. (B2) Magnified image of GCaMP6f-expressing cells in the RSP, amplified using anti-GFP antibodies. (C) Three examples of the relative change in fluorescence normalized to baseline (ΔF/F) from a rotation-sensitive RSP cell. Each trace represents a single trial. Rotation period is marked by the green box, preceded and followed by a stationary period. Below: all ΔF/F traces recorded in the cell shown above, overlaid. (D) Plot of the rate of Ca^2+^ transients recorded in the RSP cell shown in (C) during the stationary (pre-rotation) and rotation period (n = 14 trials, p = 6 × 10^−4^, Wilcoxon signed-rank test). (E) Left: average rate of transients recorded during rotation plotted against the average rate recorded during the stationary period for all cells (n = 67 cells, 6 mice). Colored circles indicate cells that showed a significant increase (green) or decrease (orange) in their event rate during rotation (dashed, unity line). Right: logarithmic plot of p values for comparison of transient rate in the stationary versus rotation period. Dashed line is the statistical significance cut-off (p = 0.05, Wilcoxon signed-rank test).

These data provide physiological evidence for head-motion signaling in V1 that currently stands in the absence of any physiologically defined pathway. To determine whether the RSP-V1 could provide a head-motion signal to L6, we took advantage of a mouse line (Ntsr1-cre) that expresses cre specifically in a subclass of L6 principal cells (Gong et al., 2007). By using a modified rabies virus (RV) (Reardon et al., 2016, Wickersham et al., 2007) (Figure 6A) to express the calcium indicator GCaMP6f (Chen et al., 2013a) in presynaptic cells (Wertz et al., 2015), we could optically record the output of RSP neurons projecting directly to V1 L6 (Figure 6B). Using two-photon (2P) microscopy while rotating the mouse in complete darkness (n = 67 cells, n = 6 mice), we found that 28% of imaged V1 L6-projecting RSP cells responded to horizontal rotation, with 16/67 showing an increase and 3/67 a decrease in activity (significance threshold p = 0.05, Wilcoxon signed-rank test; Figures 6C–6E). RSP therefore provides an anatomical and physiologically plausible pathway for the integration of head-motion signals in V1 L6 neurons.

**Figure 7:**
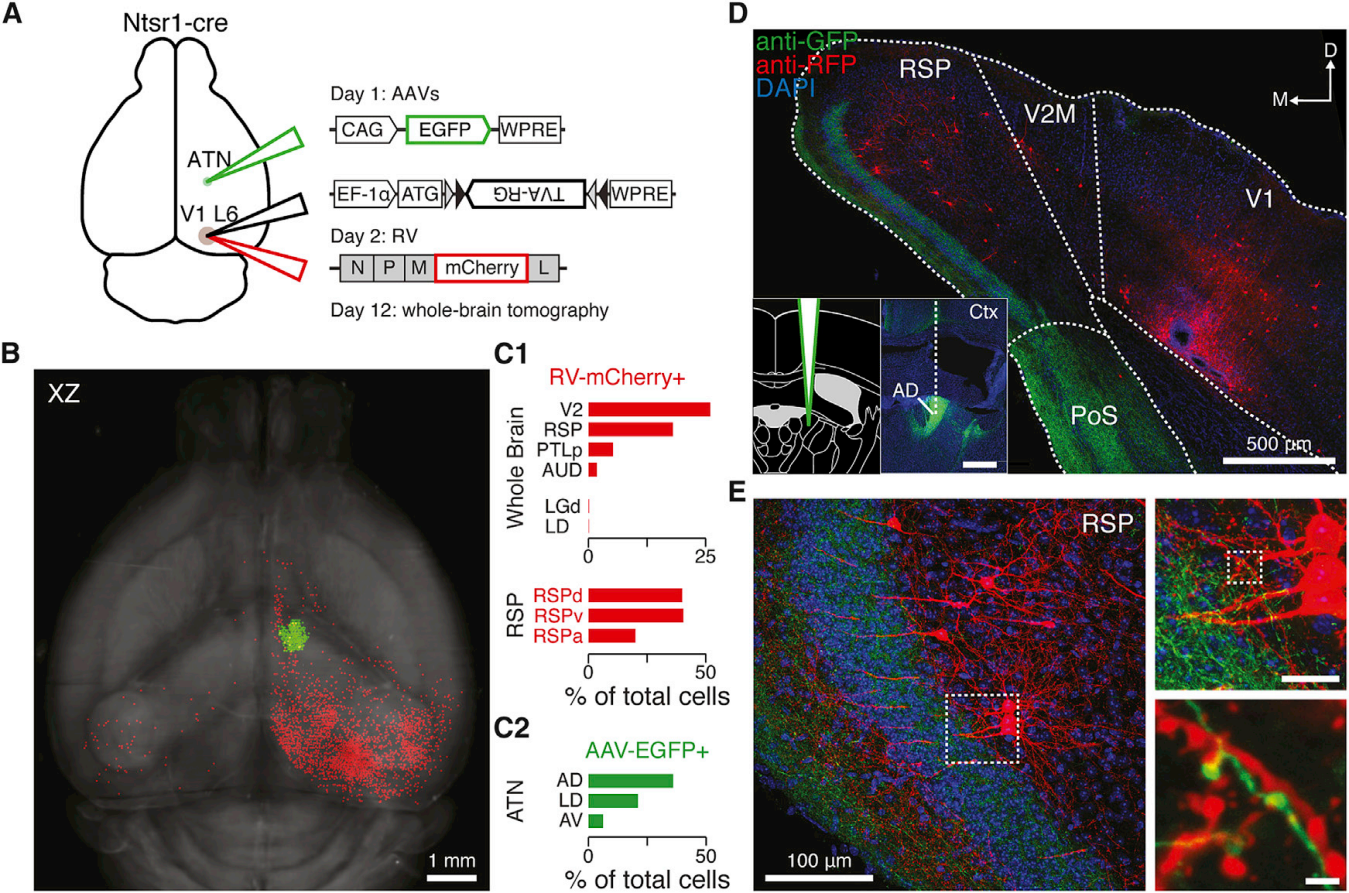
An Anatomical Pathway from RSP to V1 L6 (A) Schematic of the helper viruses encoding the EGFP (green) injected into the anterior thalamic nuclei, the TVA-RG (black), and the modified rabies virus (encoding mCherry, red) injected into V1 L6 of Ntsr1-cre mice. (B) Horizontal projection of an image stack obtained from whole-brain tomography, showing the location of cells labeled with the RV-mCherry (red) and AAV-EGFP (green). (C1) Top: bar graph showing the four most populated brain regions outside V1 containing RV-mCherry cells. V2, secondary visual cortical areas; PTLp, posterior parietal association areas; AUD, auditory areas. Middle: bar graph showing the proportion of RV-mCherry cells in the LGd (dorsal part of the lateral geniculate complex of the thalamus) and the LD (latero-dorsal nucleus of the thalamus). Below: bar graph showing the distribution of RV-mCherry cells within the RSP. RSPd, RSPv, and RSPa: dorsal, ventral, and agranular RSP, respectively. (C2) Bar graph showing the distribution of cells labeled with AAV-EGFP. ATN, anterior group of the dorsal thalamus; AD, antero-dorsal nucleus of the thalamus; LD, latero-dorsal nucleus of the thalamus; AV, antero-ventral nucleus of the thalamus. (D) Top panel: confocal coronal image of a brain slice containing both V1 and the RSP, showing the axonal projections of AAV-EGFP-expressing cells (green) and cells labeled with RV-mCherry (red). Inset: schematic (left) and coronal image (right) of the AAV1-CAGG-EGFP injection site; scale bar, 300 μm. V2M, secondary visual cortex (medial); PoS, post-subiculum. (E) Left: confocal image of the RSP showing RV-mCherry pyramidal cells with apical dendrites projecting to the granular layer, which contains dense AAV-EGFP axonal projections. Right: the area highlighted with a dashed white box, imaged at a higher magnification and showing appositions of axons and dendritic spines. Cell nuclei in these images are labeled with DAPI (blue). Scale bar, top image, 20 μm; bottom image, 2 μm.

In a final step and to further investigate whether RSP neurons presynaptic to V1 L6 cells could relay vestibular information, we targeted injections of AAV-EGFP to the anterior thalamic nuclei of Ntsr1-cre mice that had received RV injection in V1 L6 (Figure 7A). We then performed whole-brain tomography (Osten and Margrie, 2013, Ragan et al., 2012) and charted the spatial profile of AAV-EGFP- and RV mCherry-labeled neurons (Figure 7B). As previously described (Vélez-Fort et al., 2014), most RV-labeled cortical neurons outside of V1 were located in areas V2 and RSP (26% and 18%, respectively; Figure 7C). In addition, a much smaller fraction of labeled cells were also found in sub-cortical areas including the lateral geniculate nucleus (0.1%) and the anterior thalamic nuclei (ATN) (0.1%; Figure 7C). The majority of the AAV-EGFP-labeled cells were located in the ATN (63%; Figure 7C). EGFP-labeled neurons were also found in the medial group of the dorsal thalamus (MED; 27%) and the intra-laminar nuclei of the dorsal thalamus (ILM; 9%). Confocal microscopy revealed that the vast majority of RV-labeled neurons located in the RSP had pyramidal cell morphologies (Vogt and Peters, 1981). In addition, we observed a thick EGFP-labeled axon bundle projecting posterior-dorsally through layer 1 and the granular layer of the RSP (Figure 7D). Under high magnification, individual axon fibers were observed to be apposed to spines of mCherry-expressing RSP cells (Figure 7E). These data provide anatomical support of putative contacts between ascending inputs from vestibular processing areas of the thalamus and RSP cells projecting directly to V1 L6.

## Discussion

Here we report the presence of head-motion signals in deep-layer neurons of V1. In the absence of visual input, we find that the membrane potential responses of the majority of V1 L6 neurons signal the direction and angular velocity of horizontal rotation. While approximately two-thirds of responses were found to be modulated by angular velocity, other L6 neurons did not show an overall linear correlation. Qualitatively, this minority of responses appeared monotonic, and simply to signal the presence of horizontal motion rather than its speed. It remains possible that these cells could nevertheless be modulated at velocities greater than those tested here. Overall, given the abundance and amplitude of membrane potential responses found to correlate with angular velocity, and taking into account the limited fraction of Cartesian space explored (Pasquet et al., 2016, Taube, 2007), we expect head-velocity signals to have a profound role in sensory motion processing throughout V1 L6.

One challenge in studying vestibular information processing in the rodent brain is the absence of a behavioral readout that reports the detection and potential discriminability of internal motion (Angelaki et al., 2009). The psychophysical experiments performed here show that mice can discern the speed of passive rotation in darkness and that discrimination is reliant on signal transduction in the vestibular apparatus. Although we cannot say which specific features of the rotation velocity mice used to perform the task (e.g., peak versus range), this approach allowed us to generate a psychometric curve using increasingly similar stimulus pairs. These data indicate that, despite having vestibular afferent fibers displaying lower sensitivity to head velocity (Cullen, 2014), mice nevertheless exhibit relative discrimination thresholds similar to those reported by humans (Nesti et al., 2015).

These psychophysical data allowed us to directly compare the theoretical limits of velocity processing in V1 L6 to the sensory discrimination threshold of the animal. Our simulations—based on recorded subthreshold responses—indicate that a network of just 100 neurons can estimate and distinguish the speed of rotation at levels comparable with the discrimination performance of the animal. While we do not suggest that V1 L6 neurons are involved in this behavioral task, our findings indicate preservation of vestibular information signaling at the level of visual cortex. Indeed, the accuracy of motion coding by V1 L6 neurons is comparable to networks of vestibular-encoding cerebellar granule cells located one or two synapses downstream of the vestibular nucleus (Arenz et al., 2008).

In contrast to other systems in which inhibition of sensory networks has been shown to cancel self-generated internal signals (Poulet and Hedwig, 2002), we find that, during visual experience when the head is in motion, synaptic responses to internal and external motion stimuli linearly sum at the level of an individual cell. Bearing in mind that our whole-cell data show that there is no correlation between the membrane potential of L6 neurons and eye position, it is perhaps also important to highlight that, for our cross-correlation analysis, we used the average membrane potential response recorded over many trials. As such, the eye position is, on average, approximately centered for all conditions, so that the differential impact of nystagmus on average membrane potential is expected to be negligible. Given the ubiquitous nature of both rotation and visually evoked membrane potential responses, we therefore propose that visual information processing in V1 does not occur independently of head-motion signaling. In this sense, the representation of visual stimuli incorporates the motion status of the head, which can be considered a contextual element (Angelaki and Cullen, 2008) of a multisensory process.

We have also provided direct physiological evidence for a head-motion pathway onto Ntsr1^+ve^ V1 L6 cells, since presynaptic partners located in the RSP were also found to respond to head rotation under conditions of total darkness. At the population level, we observed a diversity of responses in both RSP and V1 L6 cells. Assuming head-motion signals from RSP are relayed to V1 via long-range excitatory inputs, ON and OFF responses in V1 L6 cells could at least partly be explained by our observation that different subsets of RSP cells increased or decreased their activity during rotation. Local connectivity in V1 L6 (Bortone et al., 2014, Mercer et al., 2005, West et al., 2006) could also be expected to contribute and even generate both ON and OFF responses.

In addition, we show that the axonal projections of neurons in vestibular-related thalamic nuclei (Taube, 2007) innervate the rostro-caudal axis of the RSP (Amin et al., 2010, Oh et al., 2014, Shibata, 1993, Van Groen and Wyss, 1995, Van Groen and Wyss, 2003, Vogt et al., 1981) and intersect the dendritic tree of a subset of RSP cells presynaptic to V1 L6 neurons. The presynaptic RSP population therefore contains cells that receive input from the anterior ascending vestibular pathway (Cullen and Taube, 2017). It is also conceivable that this RSP projection conveys additional information to V1 L6 (Vann et al., 2009), including contextual signals relevant to exploration and potentially to path integration.

It also remains possible that additional pathways contribute to V1 head-motion signaling. For example, in our RV tracing experiments we observed a very small proportion of presynaptic cells located in the anterior thalamic nuclei. Other sources of vestibular input could arise from multisensory brain regions, such as the secondary visual and parietal cortices (Angelaki et al., 2011, Chen et al., 2013b, Cullen and Taube, 2017), or even the lateral geniculate nucleus, where cells presynaptic to Ntsr1^+ve^ V1 L6 neurons are also found (Papaioannou, 1973, Rancz et al., 2015). However, since these brain regions also receive input from deep layers of V1 (Song et al., 2017, Vélez-Fort et al., 2014), such vestibular signals could arise from V1 feedback. Indeed, head-motion-evoked spiking activity in V1 L6 neurons will be expected to impact upper V1 cortical layers and downstream areas including the lateral geniculate nucleus (Bortone et al., 2014, Denman and Contreras, 2015, Kim et al., 2014, Olsen et al., 2012), but also the secondary visual and multisensory cortices via CC and CT projecting neurons (Vélez-Fort et al., 2014).

There is increasing evidence that V1 circuits receive top-down inputs (Muckli and Petro, 2013, Zhang et al., 2016) that relay external (Falchier et al., 2002, Fishman and Michael, 1973, Iurilli et al., 2012) and internal signals (Fu et al., 2014, Niell and Stryker, 2010, Polack et al., 2013, Saleem et al., 2013). Recent data have shown that V1 L2/3 cells can signal mismatch events when visual flow is incongruent with running speed (Keller et al., 2012). Until now the contribution of the vestibular system in generating coherent cortical sensory representations has, due to a lack of direct evidence, essentially been ignored (Klingner et al., 2016). It therefore remains unknown whether vestibular signaling in L6 could be used to detect unexpected motion events in the visual world.

In contrast to sensory-motor V1 signals, vestibular inputs would provide a direct report of the motion status of the head. We propose that, at the level of L6, motion signaling is a combinatorial representation, whereby different cells will be active depending on whether the head is being pitched, rolled, or translated. Information from the visual scene is embedded within this internal sensory-vestibular framework, which underpins a multisensory visual representation in the primary visual cortex.

## STAR★Methods

### Key Resources Table

**Table:** 

REAGENT or RESOURCE	SOURCE	IDENTIFIER
**Antibodies**		

Chicken anti-GFP	Life Technologies	RRID: AB_2534023
Rabbit anti-RFP	Rockland	RRID: AB_2209751
Goat DyLight-488 anti-chicken IgY	Abcam	RRID: AB_10681017
Donkey Alexa568 anti-rabbit IgG	Life Technologies	RRID: AB_2534017

**Bacterial and Virus Strains**		

AAV8-Flex-T-RG	Addgene	Cat# 102368
AAV8-Flex-GT	Addgene	Cat# 26198
AAV1-Flex-nGFP-2A-G	A.J. Murray	Reardon et al., 2016
CVS-N2c^ΔG^-GCaMP6f	A.J. Murray	Reardon et al., 2016
SAD-B19^ΔG^-mCherry	K.K. Conzelmann	Generous gift
AAV1-CAGG-EGFP	Addgene	Cat# 107707

**Chemicals, Peptides, and Recombinant Proteins**		

DiI	Molecular Probes	Cat# V22885
DAPI	Santa Cruz Biotechnology	CAS 28718-90-3

**Experimental Models: Cell Lines**		

N2A-N2cG cells	A.J. Murray	Reardon et al., 2016
N2A-EnvA-cytG cells	A.J. Murray	Reardon et al., 2016

**Experimental Models: Organisms/Strains**		

Mouse: C57BL/6	Charles Rivers Laboratories	N/A
Mouse: B6.FVB(Cg)-Tg(Ntsr1-cre)Gn220Gsat/Mmucd	GENSAT	RRID: MMRRC_030648-UCD

**Recombinant DNA**		

pAAV-2/1	Penn Vector Core	Cat# PL-T-PV0001
pAAV-2/8	Penn Vector Core	Cat# PL-T-PV0007

**Software and Algorithms**		

Wavemetrics Igor Pro 5	https://www.wavemetrics.com/products/igorpro/igorpro.htm	RRID: SCR_000325
Neuromatic	http://www.neuromatic.thinkrandom.com/	RRID: SCR_004186
MATLAB	https://www.mathworks.com/products/matlab.html	RRID: SCR_001622
MaSIV	https://github.com/alexanderbrown/masiv	N/A
Labview	http://www.ni.com/labview/	RRID: SCR_014325
Python 3	https://www.python.org/	RRID: SCR_008394
Pyper	https://github.com/SainsburyWellcomeCentre/Pyper	N/A
SpikeGLX	Jun et al., 2017; https://github.com/billkarsh/SpikeGLX	N/A
Kilosort	Jun et al., 2017; https://github.com/cortex-lab/KiloSort	N/A
Phy	Jun et al., 2017; https://github.com/kwikteam/phy	N/A
Turboreg plug-in in Image-J	http://bigwww.epfl.ch/thevenaz/turboreg/	RRID: SCR_014308
Custom program in C++ based on OpenCV and Pylon 5 libraries	https://opencv.org/	RRID: SCR_001905
R	http://www.r-project.org/	RRID: SCR_001905
IgorR	https://cran.r-project.org/web/packages/IgorR/index.html	N/A
aMAP	Niedworok et al., 2016; https://github.com/SainsburyWellcomeCentre/aMAP	N/A

### Contact for Reagent and Resource Sharing

Further information and requests for resources and reagents should be directed to and will be fulfilled by the Lead Contact, Troy W. Margrie (t.margrie@ucl.ac.uk).

### Experimental Model and Subject Details

#### Subjects

All experiments were performed on 6 - 15 week old black C57BL/6 or B6.FVB(Cg)-Tg(Ntsr1-cre)Gn220Gsat/Mmucd (Ntsr1-cre) mice (both male and female) in accordance with the UK Home Office regulations (Animal Welfare Act 2006) and the local animal ethics committee.

#### Viruses

SAD-B19^ΔG^-mCherry rabies virus was a generous gift of Karl-Klaus Conzelmann (Wickersham et al., 2007). CVS-N2c^ΔG^-GCaMP6f rabies virus was amplified on N2A-N2cG cells from a starter virus stock. Virus was then pseudotyped with EnvA on N2A-EnvA-cytG cells as described (Reardon et al., 2016). All cell lines and starter virus stocks were a generous gift of Andrew J. Murray. The EnvA pseudotyped virus (approximately 200ml) was purified by ultracentrifugation at 70,000 x g for 90 minutes, pellets re-suspended in Hanks Buffered Saline Solution (HBSS) and layered over 1 x HBSS 20% sucrose, centrifuged again at 50,000 x g for 90 minutes and the final pellet re-suspended in 60 μL HBSS.

SAD-B19 glycoprotein and TVA (receptor for subtype A ASLV) expressing adeno-associated virus AAV8-Flex-T-RG (Addgene number 102368), cre-dependent N2c glycoprotein expressing AAV1-Flex-nGFP-2A-G (plasmid kindly provided by Andrew J. Murray), cre-dependent TVA expressing AAV8-Flex-GT (from Addgene plasmid number 26198, a generous gift from Edward M. Callaway) and AAV1-CAGG-EGFP viruses were prepared as previously described (Vélez-Fort et al., 2014). Control experiments on all viruses and their combination were performed in non-transgenic mice. Cell culture reagents were from Sigma (Sigma Aldrich, USA), FCS from Hyclone (GE Healthcare Life Sciences, USA) and 1 x HBSS from Invitrogen (Thermo Fisher Scientific, USA).

### Method Details

#### Surgical Procedures

Unless stated otherwise, surgical procedures including the implantation of head plates, craniotomies and virus injections were carried out under either “sleep-mix,” a mixture of Fentanyl (0.05 mg/kg), midazolam (5.0 mg/kg) and medetomidine (0.5 mg/kg) in saline solution (0.9%; i.p.), or isoflurane (2%–5%). At the end of the surgery, carprofen (5mg/kg, s.c.) was typically administered and where appropriate, anesthesia was reversed with “wake-mix,” a mixture of naloxone (1.2 mg/kg), flumazenil (0.5 mg/kg) and atipamezole (2.5 mg/kg) in saline solution (0.9%; i.p.).

For bilateral lesion experiments, animals were first anesthetized with “sleep-mix” and the lateral regions of posterior and horizontal semi-circular canals were exposed. The canal bone was then thinned until punctured and a microfiber needle inserted to deliver kanamycin (5 μl; 500 mg/g b.w.). In sham animals, the temporal bone immediately adjacent to the canals was thinned and the wound closed without further intervention. Anesthesia was reversed by “wake-mix” injection and animals allowed to recover for 24 hr. All lesioned mice showed body curling when held by the base of the tail. Further vestibular deficits were quantified by recording the animals’ trajectory while exploring a circular arena using Raspberry Pi 1B, a Pi NoIR camera (30 fps) and a custom-written routine in Python (Pyper, http://www.margrie-lab.com/tools/motiontracking/). Turning was quantified by determining the cartesian coordinates of the centroid of the mouse for each frame, and calculating the change in trajectory in the horizontal plane by sequentially using coordinates from three consecutive frames. The distribution of changes in trajectory was then calculated.

For peri-ocular injections of Lidocaine, animals were first implanted with a head plate and were allowed to recover for 24 to 48 hr. Then, animals were anesthetized under isoflurane 2% and 4 × 10 μl peri-ocular injections of Lidocaine 2% were performed around each eye.

For virus injections, Ntsr1-cre mice of both sexes (8-15 weeks old) were used. Mice were anesthetized under “sleep-mix” and craniotomies performed. For 2P imaging, cre-dependent helper viruses encoding separately the TVA (AAV8-Flex-GT, 30-60 nl) and the N2c glycoprotein (AAV1-Flex-nGFP-2A-G, 60-90 nl) were injected in L6 of V1 (Bregma: −4.48 mm, ML: 2.57 mm, depth: 0.89 mm), followed 3 to 7 days later by an injection of CVS-N2c^ΔG^-GCaMP6f (120-160 nl) into the same V1 site. For whole-brain tomography, helper virus encoding both the TVA and the SAD-B19 glycoprotein protein (AAV8-Flex-T-RG, 60 nl) was stereotactically injected into L6 of V1 and, for the anatomical labeling of anterior thalamic nuclei axonal projections, an AAV virus encoding EGFP (AAV1-CAGG-EGFP, 5 nl) was injected at Bregma: −0.82 mm, ML: 0.73 mm, depth: 2.54 mm. Three days later, SAD-B19^ΔG^-mCherry (60 nl) was delivered into the same V1 site. All viruses were delivered at a rate of 1 - 2 nl/s using Nanoject III (Drummond Scientific, USA). Between nine and eleven days after rabies virus injection, a head plate was implanted for 2P imaging and the mouse allowed to recover for at least 2 days or, for whole-brain tomography, the animal was deeply anaesthetized and transcardially perfused with cold PB (0.1 M) followed by 4% paraformaldehyde (PFA) in PB (0.1 M) and the brain left overnight in 4% PFA at 4°C.

#### Sensory Stimulation

Mice were head-fixed and placed in a custom-made tube and mounted onto the recording platform fixed to a rotation motor (RV series, Newport Corporation, USA) such that left and right vestibular apparatus were positioned around the axis of rotation (Arenz et al., 2008). Mice were habituated to head fixation and rotation for 1 - 4 hours prior to recording. For electrophysiological recordings, the habituation protocol consisted of 2 sessions of 10 - 15 minutes during which the animal was rotated under normal laboratory light conditions (session 1) and then in complete darkness (session 2). For 2P imaging, the habituation consisted of a 15 - 30 minutes session during which the animal was rotated in darkness. Throughout habituation mice were given a reward consisting of condensed milk with sugar.

For visual motion stimulation, a semicircular back-projection screen was concentrically positioned 21 cm from the center of the axis of rotation of the recording platform so that a pico-projector (Picopro, Celluon, USA) mounted on a second (concentrically-positioned) motor (RV240CC, Newport Corporation, USA) could be rotated around the mouse to deliver a visual motion stimulus onto the back of the screen (100% contrast white disk, 6.6 cm in diameter). For electrophysiological recordings, a micromanipulator and head-stage was positioned on the recording platform behind the mouse and outside the field of view (behind blinkers). Horizontal rotation of the recording platform and the pico-projector was achieved using custom-written routines in Igor Pro and Neuromatic. Rotation stimuli consisted of one or two full sinusoidal periods flanked by sinusoidal ramps of the same period. Each rotation sweep was separated by 6 - 56 s of baseline. Motion stimuli were typically presented as movements of ± 90° and/or ± 180° in amplitude, reaching a maximum velocity of 80°/s. Each stimulus was repeated 4 - 15 times.

#### Extracellular Recordings

All experiments were performed on awake 8-week old black C57BL/6 mice. Whiskers were trimmed approximately 30 minutes prior to recording and the head was positioned with a pitch angle of 30° (nose down). The silicon probe was angled at 47° and the tip inserted to 1750 or 2000 μm from the pial surface. The silicon probe (IMEC, Belgium) data were acquired using an FPGA card (KC705, Xilinx, USA) and SpikeGLX (https://github.com/billkarsh/SpikeGLX) and filtered at 300 Hz. Common sources of noise were subtracted in blocks of 40 channels. Automated spike sorting was then carried out using KiloSort (https://github.com/cortex-lab/Kilosort) while implementing a 5 kHz low-pass filter, followed by manual curation of the units using Phy (http://phy-contrib.readthedocs.io/en/latest/template-gui/). Units were identified and all following analysis was carried out using routines written in Python. We excluded units with refractory period violations greater than 5% (Hill et al., 2011) and isolation distances inferior to 20 (Schmitzer-Torbert et al., 2005). Using these criteria, we were not able to confidently identify well isolated units for electrodes located in layer 2/3 (Saleem et al., 2013). Thus the data for L2/3 in Figure 1A is not considered to be single unit activity and therefore was not included in the extracellular spiking analysis. To minimize selection bias (Rossant et al., 2016) due to units whose activity was dominated by rotation-evoked firing, spontaneous and visually evoked spiking was also used. Recording sites were confirmed using DiI labeling (Molecular Probes, Thermo Fisher Scientific, USA) of the penetration track and subsequent DAPI staining (Santa Cruz Biotechnology, USA). Quantification of the spiking responses to rotation were determined on a trial by trial basis by calculating the mean spike rate recorded when the mouse was “stationary” (2 s window) and comparing it to the mean of that recorded during the entire rotation trajectory (3.5 s per direction). Wilcoxon signed-rank tests were then carried out on the means determined for all trials (and for both directions).

#### Awake Whole-cell Recordings

All experiments were performed on 6 - 12 week old black C57BL/6 mice. Whiskers were trimmed approximately 30 minutes prior to recording and the head was positioned with a pitch angle of 30° (nose down). A Mulitclamp 700B amplifier was used (Axon Instruments, USA); data were filtered at 4 kHz and digitized at 8.3 - 20 kHz using an ITC-18 ADC/DAC interface (InstruTECH, Heka Elektronik, Germany) and the Neuromatic package (http://www.neuromatic.thinkrandom.com/) under Igor Pro 5 (http://www.wavemetrics.com/). Patch pipettes were inserted through V1 at a pitch angle of 40° along the lateral-medial axis and filled with: 110 mM K-methanesulphonate, 40 mM HEPES, 6 mM NaCl, 0.02 mM CaCl2, 3 mM MgCl2, 0.05 mM EGTA, 2 mM Na2ATP, 2 mM MgATP, and 0.5 mM Na2GTP (all from Sigma Aldrich, USA); pH = 7.28 using KOH, 285 - 295 mOsm (Vapro 5520, Wescor Environmental, USA) and filtered (0.22 μm; Costar Spin-X). Recordings were obtained as previously described (Margrie et al., 2002). Recording sites were confirmed using DiI labeling (Molecular Probes, Thermo Fisher Scientific, USA) of the penetration track and subsequent DAPI staining (Santa Cruz Biotechnology, USA).

Quantification of the spiking responses to rotation were determined on a trial by trial basis by calculating the mean spike rate recorded when the mouse was “stationary” (2 s window) and comparing it to the mean of that recorded during the entire rotation trajectory (3.5 s per direction). For sub-threshold membrane potential responses, all APs were first clipped (Vélez-Fort et al., 2014) and then on a trial by trial basis the mean membrane potential recorded when the mouse was “stationary” (2 s window) was compared to the mean of that recorded during the entire rotation trajectory (3.5 s per direction). Wilcoxon signed-rank tests were then carried on the means determined for all trials (and for both directions). If a statistically significant mean response was observed for a given direction, then the evoked absolute peak amplitude for each trial (over the entire trajectory) was determined and the average calculated. For head-rotation versus visual motion comparisons, the first and second half of the rotation trajectory was used to separate “ipsi” versus “contralateral” eye field responses.

For quantifying the direction selectivity of rotation-responsive cells, direction selectivity = (Pref – Null) / (Pref + Null) where Pref = the direction showing the largest change from (stationary) baseline. For cells that showed a statistically significant depolarization in one direction and a hyperpolarization in the other (ON/OFF responses), the direction selectivity index was set to 1. To investigate the temporal dynamics of the average membrane potential, the autocorrelation of the response recorded for a given cell when the mouse was rotated past a static visual stimulus (condition 1) was first determined by comparing even and odd trials (Igor Pro 5). To compare these dynamics (condition 1) to those recorded across different experimental conditions in the same cell, cross-correlations were then performed and normalized to the peak of the autocorrelation.

The biophysical profile of recorded neurons were used to distinguish cortico-cortical and cortico-thalamic projecting L6 cells as previously described (Vélez-Fort et al., 2014). All V1 L6 regular spiking cells recorded here and in our previous work (Vélez-Fort et al., 2014) were included to generate a robust cluster tree.

#### Pupil Tracking

For pupil tracking, two cameras (acA640-750um, Basler AG, Germany) were positioned to record both ipsi and contralateral eyes at 120 fps (hardware triggered via the ITC-18) using a custom program written in C++ based on the OpenCV and Pylon 5 libraries (https://www.baslerweb.com/en/sales-support/downloads/software-downloads/). The pupil was focused using two lenses (f1 = 25 mm, f2 = 75 mm, Thorlabs, USA). To allow pupil tracking in darkness, the eyes were illuminated using an array of IR LEDs (940nm) and one drop of Pilocarpine 1% was applied topically on both eyes to reduce pupil diameter. The pupil was tracked using a development version of Pyper. The differential of the horizontal eye position over time was calculated and rapid eye movement events were detected using a threshold set to 2 - 3 x SD. Then, the average amplitude of the eye movement and of the membrane potential was calculated before and 70 ms after the onset of the eye movement event (25 ms analysis window).

#### Behavioral Experiments

All experiments were performed on 6-week old C57BL/6 mice and lasted typically 12 weeks. First, a head plate was chronically implanted and mice were allowed to recover for at least 48 hours. Mice were then given restricted access to water for 48 hours. For habituation to head restraint, mice were placed in a custom-made restriction tube and mounted onto the recording platform as described above. The platform was located within an acoustically and optically isolated chamber. The head was positioned over the center of the axis of rotation. A water reward port was positioned in front of the animal. For pre-training, the recording platform was rotated 45° in the CW direction with a peak velocity of 80°/s (S^+^ stimulus). Licking at any time 2 s after stimulus onset yielded a reward. Licking parameters were then gradually altered so that licking at the water port for two or more of five 250 ms bins, starting 2750 ms after the S^+^ stimulus onset, yielded a reward. The water reward was initiated immediately after the second bin the animal licked in. Licking for two or more bins was considered a correct response to an S^+^ stimulus. Once mice demonstrated licking in more than 80% of S^+^ trials for at least 100 trials, the S^-^ stimulus was introduced. The S^-^ stimulus was never rewarded and licking for fewer than two bins was a correct response. The percentage of correct responses was determined for each block (containing pseudo-randomly presented 10 S^+^ and 10 S^-^ trials). All stimuli had the same onset and offset times. Mice typically performed 5 - 15 blocks per day. Criterion for accurate discrimination was set at 80% on average for five consecutive blocks. The first of these five blocks were considered the first block at which an animal had performed above criterion. All mice reached criterion on the training stimulus pair within 150 blocks. Mice were then trained to discriminate odors within the rotation chamber using the same go / no-go paradigm. After treatment, 10 blocks of rotation discrimination were interleaved with 10 blocks of odor discrimination. Odors used were ethyl butyrate and cineol and performance was quantified as described above (Bracey et al., 2013).

#### Membrane Potential and Bayesian Analysis

To assess the effect of angular velocity on the evoked membrane potential, we re-sampled the membrane voltage data every 0.008°/s to ensure that every velocity value was equally represented. For each direction that showed a significant evoked response, each individual trial was shuffled according to the angular velocity while maintaining the overall temporal structure of the voltage trace. For each iteration, all shuffled trials recorded in a given cell were then averaged and a linear regression analysis was performed. The distribution of the R^2^ for 10,000 iterations (per direction) was then compared to the R^2^ of the average raw (non-shuffled) data. Thus, all p values are set at 10^−4^. The same analysis was performed to assess the contribution of acceleration, with the only difference that the membrane potential was re-sampled by taking one data point every 0.007°/s^2^.

For velocity reconstruction, we used a Bayesian approach. We combined 348 trials from eight neurons that displayed significant membrane potential responses to rotation and velocity, and for each trial computed the membrane potential as a function of (absolute) velocity. For OFF responses, the sign of the membrane potential for each trial was first corrected: ${\text{V}}_{\text{m}}^{+}=\text{sign}\left(\bar{{\text{V}}_{\text{m}}}\right){\text{V}}_{\text{m}}$ where ${\text{V}}_{\text{m}}$ is the membrane potential as a function of velocity, and $\bar{{\text{V}}_{\text{m}}}$ is the average membrane potential across all trials for a neuron and across all velocities. We then normalized all responses by scaling the minimum and maximum of each trial to [0, 1]. In the following, we considered each individual trial to have originated from a separate cell. The overall aim was to calculate the error when using different numbers of cells to reconstruct velocity. Let n be the number of cells included in the analysis. If $v_{m}=\left({\text{v}}_{1},{\text{ v}}_{2},\;\ldots ,{\text{ v}}_{\text{n}}\right)$ is the list of sign-corrected, normalized membrane potential values for each cell at a particular velocity, then Bayes’ theorem states:

$$\text{P}\left({\text{velocity| }v}_{m}\right)=\text{P}\left(v_{m}\text{ |velocity}\right)\frac{\text{P}\left(\text{velocity}\right)}{\text{P}\left(v_{m}\right)}.$$

The aim is to find: $\text{ arg }\underset{\text{velocity}}{\max}\text{P}\left({\text{velocity |}v}_{m}\right),$ which we take as a prediction of the velocity given the membrane potential observations. For theoretical convenience, we assume that the membrane potential in all cells is independent so that:

$$\text{P}\left(v_{m}\text{ |velocity}\right)=\prod_{\text{i}=1}^{\text{n}}\text{P}\left({\text{v}}_{\text{i}}|\text{velocity}\right).$$

Further, $\text{P}\left(v_{m}\right)\;$ is a scalar value and the prior P(velocity) is uniform, so that neither quantity affects the location of the peak, and velocity prediction simplifies to finding:

$$\arg\underset{\text{velocity}}{\max}\left\{\prod_{\text{i}=1}^{\text{n}}\text{P}\left({\text{v}}_{\text{i}}\text{|velocity}\right)\right\}.$$

For each cell i, we calculated the probability distribution $\text{P}\left({\text{v}}_{\text{i}}|\text{velocity}\right)$ from the n–1 other cells in the dataset. Predictions were made between 0-80°/s at 1°/s resolution. The error was defined as:

$$\text{error}=\sum_{\text{k}=1}^{80}\frac{\left|{\text{predicted velocity}}_{\text{k}}-\left(\text{k}-\frac{1}{2}\right)\right|}{80}.$$

We computed the error for different numbers of cells, ranging from 1 (single trial) to 348 (all available trials). For the 1 cell case, we computed the error for each of the 348 available trials. To compute the error when including k cells, where 1 < k < 348, we chose the k cells randomly (without replacement) from all available trials. We then repeated this random selection 348 times, to obtain a distribution of error values for a particular number of cells. The Bayesian approach was carried out in MATLAB (MathWorks, USA).

To compute the separation index, we calculated the area above the cumulative probability curve and beyond the range of the S^-^ stimulus. The area was chosen as the most appropriate measure since it is a comprehensive description of the velocity estimates falling outside the S^-^ and, second, discrimination of any stimulus from the S^-^ will likely rely on the combination of an overall increase in specific and non-specific S^+^ velocity estimates. In addition, to quantify how well 100 cells could separate the different S^+^ stimuli (S^+^2, S^+^3, S^+^4, S^+^5) from the S^-^, we estimated for one single iteration each velocity in the S^-^ range (1°/s bins, from 0 to 27°/s, giving 27 velocity estimates), and, similarly, for each velocity of the S^+^ range. Next, we determined the proportion of velocity estimates outside of the S^-^ range (> 27°/s), for both sets of estimates, and performed a Fisher’s exact test on these proportions (a p value threshold of 0.05 was used to determine significance). We performed this test for each of the 348 iterations, and for each of the S^+^ stimuli.

#### 2P Imaging

For *in vivo* GCaMP6f imaging eleven to fifteen days after rabies virus injection, mice were anaesthetized with isoflurane (2%) and a small craniotomy (1 × 2 mm) was drilled over the RSP. The craniotomy was then sealed with 2% agarose (0.1 M PB) and a 3 mm diameter round glass coverslip (#1, Harvard Apparatus, USA) and the mouse allowed to recover. At the completion of imaging, animals were deeply anaesthetized with a terminal dose of ketamine/xylazine (50:5 mg/kg) and transcardially perfused with phosphate buffer (PB 0.1 M; pH 7.2) and 4% PFA in PB (0.1 M). Following another 24 hr fixation in 4% PFA, 150 μm coronal sections were cut and slices mounted. Slices containing V1 and/or the RSP were assessed for validation of the injection site and retrograde labeling.

GCaMP6f fluorescence was imaged using a 2P moveable objective microscope (Sutter Instruments, USA) equipped with 6 mm galvo scanners (Cambridge Technology, USA) and GaAsP PMTs (Hamamatsu Photonics, Japan). Excitation laser was delivered via a Ti-Saphire laser (Insight Deepsee, Spectra Physics, USA), at 930 nm and a 16x (0.8 NA) water-immersion objective (Nikon, Japan). To minimize photodamage, the excitation laser power was adjusted to the lowest intensity required depending on the imaging depth (35 - 40 mW). Images were typically acquired from a 300 × 300 μm wide field of view at 25 frames per second and with 200 × 100 pixels sampling using custom acquisition software written in LabView (National Instruments, USA). Imaged cells were typically located 160 - 350 μm below the surface of the brain and the objective position was adjusted in x and y to align the center of the imaging plane with the center of rotation.

To de-rotate the acquired 2P images, motor position was simultaneously recorded during imaging. Briefly, the feedback output of the rotary encoder was recorded with 0.05° precision on a second imaging channel. The amount of rotation during each imaging frame was then derived from this output signal and used to shift the resized rotating frames (200 × 200 pixels) around the center of the rotation and back to the stationary position. Following de-rotation, lateral displacements were corrected using a rigid-body image registration with the Turboreg plug-in in Image-J (Thévenaz et al., 1998). ROIs were then drawn manually around the detected fluorescent somas. Fluorescent intensities in each ROI were calculated and ΔF/F traces generated with the baseline fluorescence intensity set as the mean fluorescence over the entire length of a recording sweep.

To detect calcium transients, threshold detection was performed on smoothed (median filter, kernel size = 7) and differentiated (first derivative) ΔF/F traces. Detection threshold was set at 1.2 - 1.5 SDs from the mean derivative signal. Only ROIs with at least one Ca^2+^ transient detected during the entire duration of the recording were included in the analysis. Event rate was determined for the entire duration of the rotation stimulus and compared to the same time period recorded when the platform was stationary. Statistical comparison was made using Wilcoxon signed-rank test. Custom algorithms were written in MATLAB for image de-rotation and in Python for image processing and transients detection.

#### Whole-brain Tomography and Cell Counting

For whole-brain tomography, fixed brains were embedded in 4% agar and placed under a two-photon microscope containing an integrated vibrating microtome and a motorized x-y-z stage as previously described (Vélez-Fort et al., 2014). Briefly, coronal images were acquired via three optical pathways (red, green, and blue) as a set of 6 by 9 tiles with a voxel size of 1(X) x 1(Y) μm obtained every 5 μm (Z) using an Olympus 10x objective (NA = 0.6) mounted on a piezoelectric element (Physik Instrumente, Germany). Following acquisition, image tiles were stitched and cells were manually counted and their coordinates recorded using custom image viewing software (MaSIV, https://github.com/alexanderbrown/masiv). Following this, the downscaled image stack was warped on to the Allen Brain atlas (Oh et al., 2014) using a two-step process, using an initial affine transformation followed by a second non-rigid transformation based on cubic B-splines (Niedworok et al., 2016). The resulting deformation field was used to project the detected cell positions on to the atlas. Once transformed in to atlas space, cells were assigned to brain regions according to the accompanying segmentation.

For confocal brain slice imaging, fixed brain sections (50 - 150 μm) were cut directly on a vibrating microtome (Microm HM 650V, USA) or obtained following whole-brain tomography, then rinsed in PBS. For immunostainings against GFP and mCherry, chicken anti-GFP (Life Technologies, USA, 1:500) and rabbit anti-RFP (Rockland, USA, 1:1000) primary antibodies (12 hours at room temperature) were visualized with goat DyLight-488 anti-chicken (Abcam, UK, 1:500) and donkey Alexa568 anti-rabbit (Life Technologies, USA, 1:1000) secondary antibodies (12 hours at room temperature), respectively. After thorough washing in PB, slices were mounted with a medium containing Mowiol 4-88 (Calbiochem, Germany) and DAPI (Santa Cruz Biotechnology, USA). Fluorescent images were obtained using a Leica SP8 microscope (Leica Microsystems, Germany) with 10x, 43x (oil immersion) and 63x (oil immersion) objectives.

### Quantification and Statistical Analysis

Statistical details of experiments can be found in the Results and STAR Methods sections and in figure legends, including the statistical tests used, the exact value of n and what n represents. All pooled data are represented as mean ± SEM, otherwise as indicated in the figure legends. Statistical significance level was set at p = 0.05 and the exact p value reported unless inferior to 10^−6^.

## Supplemental Information

Document S1. Figures S1–S4

Document S2. Article plus Supplemental Information

## References

[bib1] Alexander A.S., Nitz D.A. (2015). Retrosplenial cortex maps the conjunction of internal and external spaces. Nat. Neurosci..

[bib2] Amin E., Wright N., Poirier G.L., Thomas K.L., Erichsen J.T., Aggleton J.P. (2010). Selective lamina dysregulation in granular retrosplenial cortex (area 29) after anterior thalamic lesions: an in situ hybridization and trans-neuronal tracing study in rats. Neuroscience.

[bib3] Angelaki D.E., Cullen K.E. (2008). Vestibular system: the many facets of a multimodal sense. Annu. Rev. Neurosci..

[bib4] Angelaki D.E., Gu Y., DeAngelis G.C. (2009). Multisensory integration: psychophysics, neurophysiology, and computation. Curr. Opin. Neurobiol..

[bib5] Angelaki D.E., Gu Y., Deangelis G.C. (2011). Visual and vestibular cue integration for heading perception in extrastriate visual cortex. J. Physiol..

[bib6] Arenz A., Silver R.A., Schaefer A.T., Margrie T.W. (2008). The contribution of single synapses to sensory representation in vivo. Science.

[bib7] Barlow H.B., Levick W.R. (1965). The mechanism of directionally selective units in rabbit’s retina. J. Physiol..

[bib8] Barlow H.B., Blakemore C., Pettigrew J.D. (1967). The neural mechanism of binocular depth discrimination. J. Physiol..

[bib9] Bortone D.S., Olsen S.R., Scanziani M. (2014). Translaminar inhibitory cells recruited by layer 6 corticothalamic neurons suppress visual cortex. Neuron.

[bib10] Bracey E.F., Pichler B., Schaefer A.T., Wallace D.J., Margrie T.W. (2013). Perceptual judgements and chronic imaging of altered odour maps indicate comprehensive stimulus template matching in olfaction. Nat. Commun..

[bib11] Chen L.L., Lin L.H., Green E.J., Barnes C.A., McNaughton B.L. (1994). Head-direction cells in the rat posterior cortex. I. Anatomical distribution and behavioral modulation. Exp. Brain Res..

[bib12] Chen T.-W., Wardill T.J., Sun Y., Pulver S.R., Renninger S.L., Baohan A., Schreiter E.R., Kerr R.A., Orger M.B., Jayaraman V. (2013). Ultrasensitive fluorescent proteins for imaging neuronal activity. Nature.

[bib13] Chen X., Deangelis G.C., Angelaki D.E. (2013). Diverse spatial reference frames of vestibular signals in parietal cortex. Neuron.

[bib14] Cho J., Sharp P.E. (2001). Head direction, place, and movement correlates for cells in the rat retrosplenial cortex. Behav. Neurosci..

[bib15] Cullen K.E. (2014). The neural encoding of self-generated and externally applied movement: implications for the perception of self-motion and spatial memory. Front. Integr. Nuerosci..

[bib16] Cullen K.E., Taube J.S. (2017). Our sense of direction: progress, controversies and challenges. Nat. Neurosci..

[bib17] Denman D.J., Contreras D. (2015). Complex effects on in vivo visual responses by specific projections from mouse cortical layer 6 to dorsal lateral geniculate nucleus. J. Neurosci..

[bib18] Dräger U.C. (1975). Receptive fields of single cells and topography in mouse visual cortex. J. Comp. Neurol..

[bib19] Erisken S., Vaiceliunaite A., Jurjut O., Fiorini M., Katzner S., Busse L. (2014). Effects of locomotion extend throughout the mouse early visual system. Curr. Biol..

[bib20] Falchier A., Clavagnier S., Barone P., Kennedy H. (2002). Anatomical evidence of multimodal integration in primate striate cortex. J. Neurosci..

[bib21] Fiser A., Mahringer D., Oyibo H.K., Petersen A.V., Leinweber M., Keller G.B. (2016). Experience-dependent spatial expectations in mouse visual cortex. Nat. Neurosci..

[bib22] Fishman M.C., Michael P. (1973). Integration of auditory information in the cat’s visual cortex. Vision Res..

[bib23] Fu Y., Tucciarone J.M., Espinosa J.S., Sheng N., Darcy D.P., Nicoll R.A., Huang Z.J., Stryker M.P. (2014). A cortical circuit for gain control by behavioral state. Cell.

[bib24] Gong S., Doughty M., Harbaugh C.R., Cummins A., Hatten M.E., Heintz N., Gerfen C.R. (2007). Targeting Cre recombinase to specific neuron populations with bacterial artificial chromosome constructs. J. Neurosci..

[bib25] Grossman E.D., Blake R. (2002). Brain areas active during visual perception of biological motion. Neuron.

[bib26] Grüsser O.J., Grüsser-Cornehls U. (1972). Interaction of vestibular and visual inputs in the visual system. Prog. Brain Res..

[bib27] Hill D.N., Mehta S.B., Kleinfeld D. (2011). Quality metrics to accompany spike sorting of extracellular signals. J. Neurosci..

[bib28] Hoy J.L., Yavorska I., Wehr M., Niell C.M. (2016). Vision drives accurate approach behavior during prey capture in laboratory mice. Curr. Biol..

[bib29] Hubel D.H. (1960). Single unit activity in lateral geniculate body and optic tract of unrestrained cats. J. Physiol..

[bib30] Hubel D.H., Wiesel T.N. (1959). Receptive fields of single neurones in the cat’s striate cortex. J. Physiol..

[bib31] Hubel D.H., Wiesel T.N. (1968). Receptive fields and functional architecture of monkey striate cortex. J. Physiol..

[bib32] Iurilli G., Ghezzi D., Olcese U., Lassi G., Nazzaro C., Tonini R., Tucci V., Benfenati F., Medini P. (2012). Sound-driven synaptic inhibition in primary visual cortex. Neuron.

[bib33] Jacob P.Y., Casali G., Spieser L., Page H., Overington D., Jeffery K. (2017). An independent, landmark-dominated head-direction signal in dysgranular retrosplenial cortex. Nat. Neurosci..

[bib34] Jun J.J., Steinmetz N.A., Siegle J.H., Denman D.J., Bauza M., Barbarits B., Lee A.K., Anastassiou C.A., Andrei A., Aydın Ç. (2017). Fully integrated silicon probes for high-density recording of neural activity. Nature.

[bib35] Keller G.B., Bonhoeffer T., Hübener M. (2012). Sensorimotor mismatch signals in primary visual cortex of the behaving mouse. Neuron.

[bib36] Kim J., Matney C.J., Blankenship A., Hestrin S., Brown S.P. (2014). Layer 6 corticothalamic neurons activate a cortical output layer, layer 5a. J. Neurosci..

[bib37] Klingner C.M., Axer H., Brodoehl S., Witte O.W. (2016). Vertigo and the processing of vestibular information: a review in the context of predictive coding. Neurosci. Biobehav. Rev..

[bib38] Leinweber M., Ward D.R., Sobczak J.M., Attinger A., Keller G.B. (2017). A sensorimotor circuit in mouse cortex for visual flow predictions. Neuron.

[bib39] Makino H., Komiyama T. (2015). Learning enhances the relative impact of top-down processing in the visual cortex. Nat. Neurosci..

[bib40] Mao D., Kandler S., McNaughton B.L., Bonin V. (2017). Sparse orthogonal population representation of spatial context in the retrosplenial cortex. Nat. Commun..

[bib41] Margrie T.W., Brecht M., Sakmann B. (2002). In vivo, low-resistance, whole-cell recordings from neurons in the anaesthetized and awake mammalian brain. Pflugers Arch..

[bib42] Mercer A., West D.C., Morris O.T., Kirchhecker S., Kerkhoff J.E., Thomson A.M. (2005). Excitatory connections made by presynaptic cortico-cortical pyramidal cells in layer 6 of the neocortex. Cereb. Cortex.

[bib43] Morris R.G., Garrud P., Rawlins J.N., O’Keefe J. (1982). Place navigation impaired in rats with hippocampal lesions. Nature.

[bib44] Moser E.I., Kropff E., Moser M.B. (2008). Place cells, grid cells, and the brain’s spatial representation system. Annu. Rev. Neurosci..

[bib45] Muckli L., Petro L.S. (2013). Network interactions: non-geniculate input to V1. Curr. Opin. Neurobiol..

[bib46] Nesti A., Beykirch K.A., Pretto P., Bülthoff H.H. (2015). Human discrimination of head-centred visual-inertial yaw rotations. Exp. Brain Res..

[bib47] Niedworok C.J., Brown A.P.Y., Jorge Cardoso M., Osten P., Ourselin S., Modat M., Margrie T.W. (2016). aMAP is a validated pipeline for registration and segmentation of high-resolution mouse brain data. Nat. Commun..

[bib48] Niell C.M. (2015). Cell types, circuits, and receptive fields in the mouse visual cortex. Annu. Rev. Neurosci..

[bib49] Niell C.M., Stryker M.P. (2010). Modulation of visual responses by behavioral state in mouse visual cortex. Neuron.

[bib50] Oh S.W., Harris J.A., Ng L., Winslow B., Cain N., Mihalas S., Wang Q., Lau C., Kuan L., Henry A.M. (2014). A mesoscale connectome of the mouse brain. Nature.

[bib51] Olsen S.R., Bortone D.S., Adesnik H., Scanziani M. (2012). Gain control by layer six in cortical circuits of vision. Nature.

[bib52] Osten P., Margrie T.W. (2013). Mapping brain circuitry with a light microscope. Nat. Methods.

[bib53] Papaioannou J.N. (1973). Effects of caloric labyrinthine stimulation on the spontaneous activity of lateral geniculate nucleus neurons in the cat. Exp. Brain Res..

[bib54] Pasquet M.O., Tihy M., Gourgeon A., Pompili M.N., Godsil B.P., Léna C., Dugué G.P. (2016). Wireless inertial measurement of head kinematics in freely-moving rats. Sci. Rep..

[bib55] Polack P.-O., Friedman J., Golshani P. (2013). Cellular mechanisms of brain state-dependent gain modulation in visual cortex. Nat. Neurosci..

[bib56] Poort J., Khan A.G., Pachitariu M., Nemri A., Orsolic I., Krupic J., Bauza M., Sahani M., Keller G.B., Mrsic-Flogel T.D., Hofer S.B. (2015). Learning enhances sensory and multiple non-sensory representations in primary visual cortex. Neuron.

[bib57] Poulet J.F., Hedwig B. (2002). A corollary discharge maintains auditory sensitivity during sound production. Nature.

[bib58] Ragan T., Kadiri L.R., Venkataraju K.U., Bahlmann K., Sutin J., Taranda J., Arganda-Carreras I., Kim Y., Seung H.S., Osten P. (2012). Serial two-photon tomography for automated ex vivo mouse brain imaging. Nat. Methods.

[bib59] Rancz E.A., Moya J., Drawitsch F., Brichta A.M., Canals S., Margrie T.W. (2015). Widespread vestibular activation of the rodent cortex. J. Neurosci..

[bib60] Reardon T.R., Murray A.J., Turi G.F., Wirblich C., Croce K.R., Schnell M.J., Jessell T.M., Losonczy A. (2016). Rabies virus CVS-N2c(ΔG) strain enhances retrograde synaptic transfer and neuronal viability. Neuron.

[bib61] Roelfsema P.R., Lamme V.A., Spekreijse H. (1998). Object-based attention in the primary visual cortex of the macaque monkey. Nature.

[bib62] Rossant C., Kadir S.N., Goodman D.F.M., Schulman J., Hunter M.L.D., Saleem A.B., Grosmark A., Belluscio M., Denfield G.H., Ecker A.S. (2016). Spike sorting for large, dense electrode arrays. Nat. Neurosci..

[bib63] Roth M.M., Helmchen F., Kampa B.M. (2012). Distinct functional properties of primary and posteromedial visual area of mouse neocortex. J. Neurosci..

[bib64] Roth M.M., Dahmen J.C., Muir D.R., Imhof F., Martini F.J., Hofer S.B. (2016). Thalamic nuclei convey diverse contextual information to layer 1 of visual cortex. Nat. Neurosci..

[bib65] Saleem A.B., Ayaz A., Jeffery K.J., Harris K.D., Carandini M. (2013). Integration of visual motion and locomotion in mouse visual cortex. Nat. Neurosci..

[bib66] Schmitzer-Torbert N., Jackson J., Henze D., Harris K., Redish A.D. (2005). Quantitative measures of cluster quality for use in extracellular recordings. Neuroscience.

[bib67] Shibata H. (1993). Efferent projections from the anterior thalamic nuclei to the cingulate cortex in the rat. J. Comp. Neurol..

[bib68] Smith P.F., Horii A., Russell N., Bilkey D.K., Zheng Y., Liu P., Kerr D.S., Darlington C.L. (2005). The effects of vestibular lesions on hippocampal function in rats. Prog. Neurobiol..

[bib69] Song Y.H., Kim J.H., Jeong H.W., Choi I., Jeong D., Kim K., Lee S.H. (2017). A neural circuit for auditory dominance over visual perception. Neuron.

[bib70] Stackman R.W., Clark A.S., Taube J.S. (2002). Hippocampal spatial representations require vestibular input. Hippocampus.

[bib71] Taube J.S. (2007). The head direction signal: origins and sensory-motor integration. Annu. Rev. Neurosci..

[bib72] Taube J.S., Muller R.U., Ranck J.B. (1990). Head-direction cells recorded from the postsubiculum in freely moving rats. II. Effects of environmental manipulations. J. Neurosci..

[bib73] Thévenaz P., Ruttimann U.E., Unser M. (1998). A pyramid approach to subpixel registration based on intensity. IEEE Trans. Image Process..

[bib74] Thiel A., Greschner M., Eurich C.W., Ammermüller J., Kretzberg J. (2007). Contribution of individual retinal ganglion cell responses to velocity and acceleration encoding. J. Neurophysiol..

[bib75] Toyama K., Komatsu Y., Shibuki K. (1984). Integration of retinal and motor signals of eye movements in striate cortex cells of the alert cat. J. Neurophysiol..

[bib76] Tsoar A., Nathan R., Bartan Y., Vyssotski A., Dell’Omo G., Ulanovsky N. (2011). Large-scale navigational map in a mammal. Proc. Natl. Acad. Sci. USA.

[bib77] Valerio S., Taube J.S. (2016). Head direction cell activity is absent in mice without the horizontal semicircular canals. J. Neurosci..

[bib78] Van Essen D.C., Gallant J.L. (1994). Neural mechanisms of form and motion processing in the primate visual system. Neuron.

[bib79] van Groen T., Wyss J.M. (1992). Connections of the retrosplenial dysgranular cortex in the rat. J. Comp. Neurol..

[bib80] Van Groen T., Wyss J.M. (1995). Projections from the anterodorsal and anteroventral nucleus of the thalamus to the limbic cortex in the rat. J. Comp. Neurol..

[bib81] Van Groen T., Wyss J.M. (2003). Connections of the retrosplenial granular b cortex in the rat. J. Comp. Neurol..

[bib82] Vann S.D., Aggleton J.P., Maguire E.A. (2009). What does the retrosplenial cortex do?. Nat. Rev. Neurosci..

[bib83] Vanni-Mercier G., Magnin M. (1982). Single neuron activity related to natural vestibular stimulation in the cat’s visual cortex. Exp. Brain Res..

[bib84] Vélez-Fort M., Rousseau C.V., Niedworok C.J., Wickersham I.R., Rancz E.A., Brown A.P.Y., Strom M., Margrie T.W. (2014). The stimulus selectivity and connectivity of layer six principal cells reveals cortical microcircuits underlying visual processing. Neuron.

[bib85] Vidal P.-P., Degallaix L., Josset P., Gasc J.-P., Cullen K.E. (2004). Postural and locomotor control in normal and vestibularly deficient mice. J. Physiol..

[bib86] Vogt B.A., Miller M.W. (1983). Cortical connections between rat cingulate cortex and visual, motor, and postsubicular cortices. J. Comp. Neurol..

[bib87] Vogt B.A., Peters A. (1981). Form and distribution of neurons in rat cingulate cortex: areas 32, 24, and 29. J. Comp. Neurol..

[bib88] Vogt B.A., Rosene D.L., Peters A. (1981). Synaptic termination of thalamic and callosal afferents in cingulate cortex of the rat. J. Comp. Neurol..

[bib89] Wertz A., Trenholm S., Yonehara K., Hillier D., Raics Z., Leinweber M., Szalay G., Ghanem A., Keller G., Rózsa B. (2015). Presynaptic networks. Single-cell-initiated monosynaptic tracing reveals layer-specific cortical network modules. Science.

[bib90] West D.C., Mercer A., Kirchhecker S., Morris O.T., Thomson A.M. (2006). Layer 6 cortico-thalamic pyramidal cells preferentially innervate interneurons and generate facilitating EPSPs. Cereb. Cortex.

[bib91] Wickersham I.R., Lyon D.C., Barnard R.J.O., Mori T., Finke S., Conzelmann K.-K., Young J.A.T., Callaway E.M. (2007). Monosynaptic restriction of transsynaptic tracing from single, genetically targeted neurons. Neuron.

[bib92] Yan Y., Rasch M.J., Chen M., Xiang X., Huang M., Wu S., Li W. (2014). Perceptual training continuously refines neuronal population codes in primary visual cortex. Nat. Neurosci..

[bib93] Zhang S., Xu M., Kamigaki T., Hoang Do J.P., Chang W.-C., Jenvay S., Miyamichi K., Luo L., Dan Y. (2014). Selective attention. Long-range and local circuits for top-down modulation of visual cortex processing. Science.

[bib94] Zhang S., Xu M., Chang W.-C., Ma C., Hoang Do J.P., Jeong D., Lei T., Fan J.L., Dan Y. (2016). Organization of long-range inputs and outputs of frontal cortex for top-down control. Nat. Neurosci..

