# Supplementary material for: A Circuit for Integration of Head- and Visual-Motion Signals in Layer 6 of Mouse Primary Visual Cortex
Source: Neuron. 2018 Apr 4;98(1):179–191.e6. doi: 10.1016/j.neuron.2018.02.023 (PMC5896233; doi:10.1016/j.neuron.2018.02.023)
Supplement: Document S1. Figures S1–S4 [file mmc1.pdf]

Neuron, Volume *98*

Supplemental Information

A Circuit for Integration of Head- and Visual-Motion Signals in Layer 6 of Mouse Primary Visual Cortex

Mateo Vélez-Fort, Edward F. Bracey, Sepiedeh Keshavarzi, Charly V. Rousseau, Lee Cossell, Stephen C. Lenzi, Molly Strom, and Troy W. Margrie

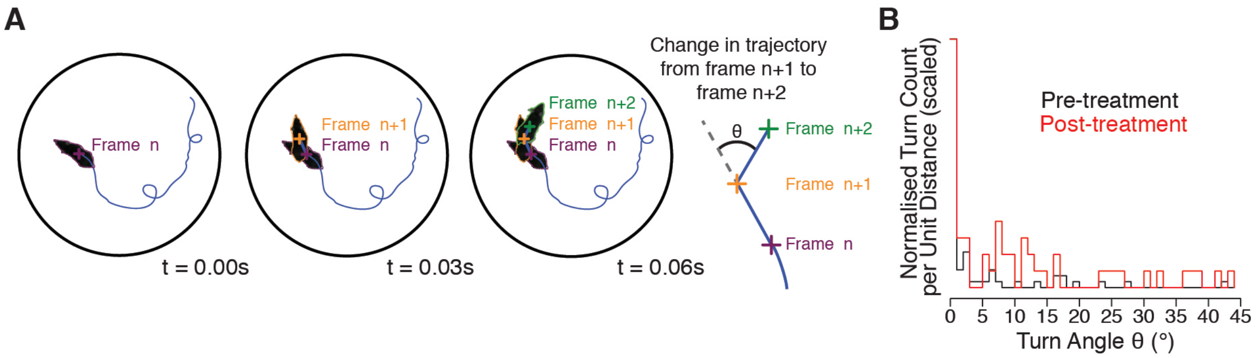

Figure S1. Related to Figure 1. Locomotion trajectories in control and vestibular-lesioned mice
(A) Schematic of three successive frames recorded during exploration of a circular arena (40 cm diameter). The computed centroids of the animal are shown for each frame (colored crosses). Right, schematic of the calculation of the turn angle for the example trajectory shown in the previous three frames.
(B) Normalized and scaled histogram of the turn angles (per unit distance, bin size = 1°) for four mice, prior to (black) and following (red) vestibular lesioning (significance threshold $p = 0.05$, KS test).

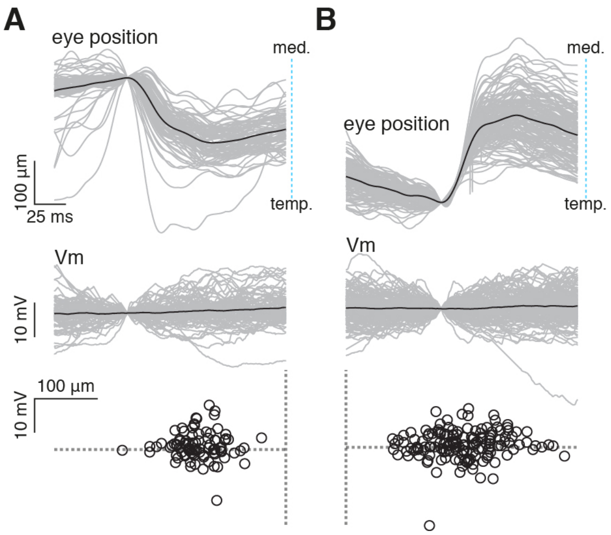

Figure S2. Related to Figure 1. Eye position and membrane potential of L6 neurons recorded during rotation

(A) Top, individual (87 events, grey) and average (black) traces of the medial to temporal repositioning of the eye recorded during rotation. Middle, membrane potential traces (grey) overlaid with the average (black) simultaneously recorded from one L6 cell. Bottom, plot of the average eye position (determined from a 25 ms window, 70 ms after movement onset) against the average membrane potential recorded over the same time window (significance threshold p = 0.05, Wilcoxon signed-rank test).

(B) Top, individual (149 events, grey) and average (black) traces of the temporal to medial repositioning of the eye recorded during rotation. Middle, membrane potential traces (grey) overlaid with the average (black) simultaneously recorded from a L6 cell. Bottom, the change in eye position along the same axis (for each event) quantified over a 25 ms time window 70 ms after movement onset plotted against the change (from pre-movement baseline) in the membrane potential during the same analysis time window (significance threshold p = 0.05, Wilcoxon signed-rank test; n = 6 trajectories, 3 mice).

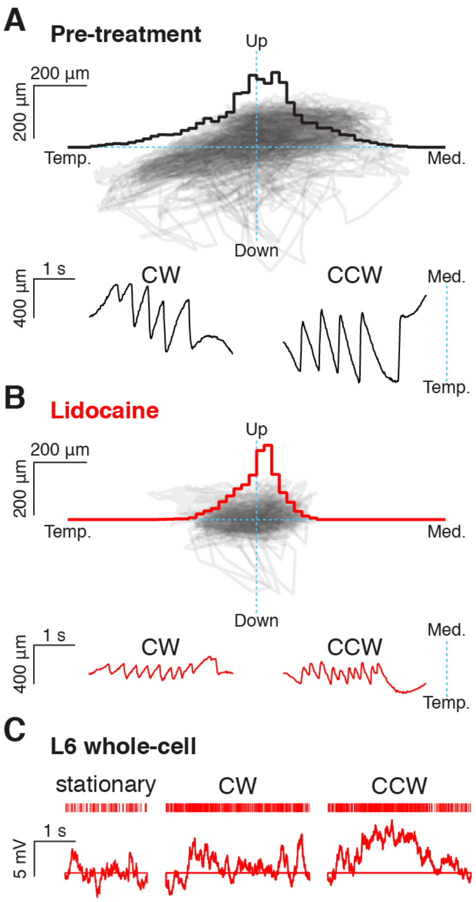

Figure S3. Related to Figure 1. Rotation-evoked responses in L6 neurons following peri-ocular injections of Lidocaine

(A) Top, a trace of the pupil position recorded over the duration of a typical experiment (grey trace, 4-5 minutes recording). Overlaid is the normalized histogram of eye position along the horizontal axis (black). Below, traces showing the pupil position during rotation in the CW and CCW directions, plotted over time.

(B) Top, a trace of the position of the pupil shown in (A) following injection of Lidocaine (grey trace, 4-5 minutes recording). Overlaid is the normalized histogram of eye position along the horizontal axis (red). Below, traces showing the pupil position during rotation in the CW and CCW directions, plotted over time (control SD = 13.75 ± 0.59 versus Lidocaine SD = 5.44 ± 1.07, significance threshold p = 0.05, Wilcoxon signed-rank test; n = 6 trajectories, 3 mice).

(C) Top, raster plot of spiking obtained from a whole-cell recording of a L6 neuron after peri-ocular injection of Lidocaine in the absence and presence of rotation. Below, the average membrane potential recorded in the same cell. Horizontal line indicates the mean membrane potential recorded in the absence of rotation.

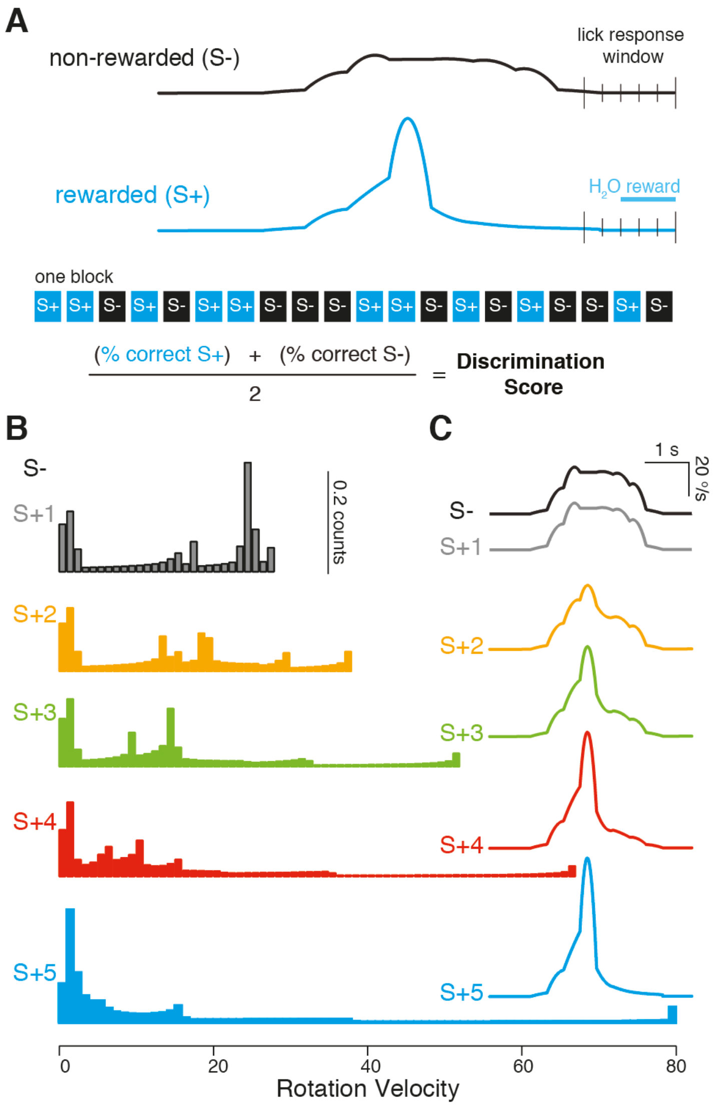

Figure S4. Related to Figure 2. Paradigm for assessing vestibular-mediated discrimination of rotation stimuli

(A) Top, schematic of individual non-rewarded (S^-) and rewarded (S^+) trials indicating the response window at the end of each trial. The response to the S^- is considered correct if the mouse fails to lick or licks in only one bin. Responses to the S^+ are considered correct when the mouse licks in at least in 2 bins. Bottom, schematic showing the composition of a single block of trials (10 S^+ and 10 S^- trials presented in pseudo-random fashion) used to calculate discrimination accuracy scores.

(B) Histograms of the velocity of the six rotation stimuli used in the discrimination task.

(C) Examples of the velocity profile of the rotation stimuli used in the discrimination task. Note S^- and S^+1 are identical.
